# Sas3 and Ada2(Gcn5)-dependent histone H3 acetylation is required for transcription elongation at the de-repressed *FLO1* gene

**DOI:** 10.1093/nar/gkx028

**Published:** 2017-01-23

**Authors:** Michael Church, Kim C. Smith, Mohamed M. Alhussain, Sari Pennings, Alastair B. Fleming

**Affiliations:** 1 School of Genetics and Microbiology, University of Dublin, Trinity College Dublin, College Green, Dublin 2, Ireland; 2 Queen's Medical Research Institute, University of Edinburgh, Edinburgh, EH16 4TJ, UK

## Abstract

The *Saccharomyces cerevisiae FLO1* gene encodes a cell wall protein that imparts cell–cell adhesion. *FLO1* transcription is regulated via the antagonistic activities of the Tup1–Cyc8 co-repressor and Swi–Snf co-activator complexes. Tup1–Cyc8 represses transcription through the organization of strongly positioned, hypoacetylated nucleosomes across gene promoters. Swi–Snf catalyzes remodeling of these nucleosomes in a mechanism involving histone acetylation that is poorly understood. Here, we show that *FLO1* de-repression is accompanied by Swi–Snf recruitment, promoter histone eviction and Sas3 and Ada2(Gcn5)-dependent histone H3K14 acetylation. In the absence of H3K14 acetylation, Swi–Snf recruitment and histone eviction proceed, but transcription is reduced, suggesting these processes, while essential, are not sufficient for de-repression. Further analysis in the absence of H3K14 acetylation reveals RNAP II recruitment at the *FLO1* promoter still occurs, but RNAP II is absent from the gene-coding region, demonstrating Sas3 and Ada2-dependent histone H3 acetylation is required for transcription elongation. Analysis of the transcription kinetics at other genes reveals shared mechanisms coupled to a distinct role for histone H3 acetylation, essential at *FLO1*, downstream of initiation. We propose histone H3 acetylation in the coding region provides rate-limiting control during the transition from initiation to elongation which dictates whether the gene is permissive for transcription.

## INTRODUCTION

The yeast *FLO1* gene encodes a lectin-like cell wall protein, which promotes non-sexual, calcium-dependent cell aggregation observable as a flocculation phenotype (1–3). *FLO1* is the dominant member of a family of *FLO* genes, which includes *FLO5, FLO9* and *FLO10* (4). Flocculation provides cell populations with a survival strategy against external stresses whereby cells within the ‘floc’ are physically shielded from the outside environment (5). Flocculation has also been shown to enhance cell mating (6). Thus, flocculation is an important phenotype by which populations of cells collaborate to aid their mutual survival. This phenotype is important in biofilm formation, and in industries such as brewing where it aids in the removal of yeast cells after fermentation (7,8).

Under nutrient rich conditions, the *FLO1* gene is repressed by the Tup1–Cyc8(Ssn6) co-repressor complex (9,10). Tup1–Cyc8 was the first co-repressor to be identified and functions to repress genes involved in many cellular processes, including genes regulated by glucose, oxygen, mating type and DNA damage (11–13). The Tup1–Cyc8 complex does not bind DNA directly, but is recruited to target genes by DNA binding proteins such as Mig1 at the glucose repressed *SUC2* gene, and Sko1 at the osmotic stress response genes (14–16). Tup1–Cyc8 has been proposed to repress target genes via a number of mechanisms including; (i) the establishment of a highly ordered nucleosomal array; (ii) recruitment of histone deacetylases (HDACs) to promote histone deacetylation; (iii) direct inhibition of RNA polymerase II (RNAP II) recruitment and (iv) the exclusion of activator proteins (17–26). These different mechanisms may function depending on the gene target, and are not mutually exclusive (27–29).

Our previous studies have shown that Tup1–Cyc8 cooperates with the HDACs Rpd3 and Hda1 to repress *FLO1* transcription via the establishment of an extensive array of highly ordered, hypoacetylated nucleosomes across the *FLO1* promoter and upstream region (26,30). In the absence of Tup1–Cyc8, *FLO1* de-repression is accompanied by histone acetylation and a gross disruption of the promoter chromatin which includes extensive nucleosome repositioning and loss (13,26,30). The Swi–Snf complex was implicated in this nucleosome rearrangement and *FLO1* gene de-repression, since in a *cyc8 snf2* double mutant, both remodeling and *FLO1* transcription are absent (26). Thus, *FLO1* transcription is regulated via its promoter chromatin which is under the antagonistic control of the Tup1–Cyc8 co-repressor and the Swi–Snf co-activator complexes.

Although the mechanism of *FLO1* gene repression by Tup1–Cyc8 has been examined in detail, little is known about events during de-repression in the absence of Tup1–Cyc8. The main histone acetyltransferases (HATs) associated with transcription in yeast are Gcn5, Sas3 and Esa1 (31). Gcn5 resides in the SAGA, ADA and SLIK complexes and catalyzes the acetylation of H3 and H2B, while the NuA3 complex subunit Sas3, and the NuA4 complex component Esa1, acetylate H3 and H4 respectively. Depending on the gene target, the acetylation marks can be present in the promoter or gene coding regions where they can function by recruiting non histone proteins to further influence chromatin structure and function. As an example of interdependence between epigenetic marks, H3 lysine 14 acetylation (H3K14ac) is required for Set1-dependent H3 lysine 4 trimethylation (H3K4me3), which in turn binds the NuA3 complex promoting Sas3-dependent H3K14ac (32–34). Thus in yeast, Gcn5/NuA3-dependent H3K14ac can be considered a primary upstream mark of transcription found at many active gene promoters.

In this study, we wanted to investigate which HATs were required for *FLO1* de-repression, and to determine their role in this process. Our data revealed that in the absence of Tup1–Cyc8, Gcn5 and Sas3-dependent acetylation of lysine 14 of histone H3 at the *FLO1* promoter and ORF was required for *FLO1* transcription. We found that Swi–Snf recruitment and histone eviction at the *FLO1* promoter were not dependent upon Ada2(Gcn5) and Sas3 mediated histone acetylation, and that histone eviction does not in itself enable *FLO1* transcription. Interestingly we found that following depletion of Cyc8 from the nucleus using the anchor-away technique, de-repression of the flocculation phenotype occurred via the biphasic recruitment of RNAP II to the *FLO1* gene and the gradual accumulation of *FLO1* mRNA. Furthermore we discovered that Ada2 and Sas3 are not required for RNAP II recruitment to the *FLO1* promoter but occupancy of RNAP II in the *FLO1* open reading frame (ORF) is Ada2(Gcn5) and Sas3-dependent. These data are consistent with a model whereby Sas3 and Ada2-dependent HAT acetylation of histone H3 lysine 14 is required for RNAP II elongation at the de-repressed *FLO1* gene.

## MATERIALS AND METHODS

### Yeast strains

The *Saccharomyces cerevisiae* strains used are described in Supplementary Table S1. The histone mutant strains were a generous gift from Mary Ann Osley (Supplementary Table S1). Yeast gene deletions and tagging were performed using polymerase chain reaction (PCR)-based methods (35,36). All gene deletions were confirmed by PCR or western blot analysis and assayed for the appropriate phenotypes. PCR and western blot analysis were used to confirm that the genomic copies of *GCN5* and *SAS3* were correctly tagged with a C-terminal nine Myc epitope (Supplementary Figure S1A–C). Epitope tagged strains were assayed to confirm appropriate wild-type (wt) phenotypes. The Cyc8 anchor-away strain was constructed as described previously, and confirmed by western blot and chromatin immunoprecipitation (ChIP) analysis (Supplementary Figure S2) (37). Cells were grown in YEPD medium at 30°C unless otherwise stated.

### RNA analysis

RNA extraction, cDNA preparation and RT-qPCR analysis were performed as previously described (30). Values were normalized to *ACT1* RNA. Primers used are listed in Supplementary Table S2.

### Protein analysis

Protein lysate preparation and western blot analysis were performed as previously described (30). The antibodies and conditions used are described in Supplementary Table S3.

### Anchor-away experiments

The anchor-away protocol was performed as previously described (37). Cells were first grown overnight in YEPD medium at 30°C and then diluted in fresh YEPD and grown until OD_600_ ∼0.4. A sample was then taken (time 0), after which anchor-away was induced by the addition of rapamycin (Fisher) to the remaining culture at a final concentration of 1 μg/ml. Samples were removed at the times indicated and either cross-linked for ChIP analysis, or left untreated for RNA preparation. Reaction kinetics observed following anchor-away depletion were affected by rate-limiting availability of various components *in vivo*, and did not allow for rate constant analysis in this study.

### Chromatin immunoprecipitation (ChIP)

ChIP was performed as previously described (30,38). The antibodies and conditions used are shown in Supplementary Table S4. The anti-Snf2 and anti-Tup1 antibodies were generous gifts from J. Reese. The IP/input ratio for target sequences were normalized to the IP/input ratio at *TEL-VI* (RNAP II, H4ac4, H3K9ac, H3K14ac, Snf2), *INT-V* (H3) or *ACT1* (Gcn5-Myc, Sas3-Myc) sequences. Histone acetylation levels were further normalized relative to the corresponding histone H3 levels at each site. For Tup1 ChIP analysis during steady state and anchor-away experiments, occupancy was expressed as the IP/in ratio for *FLO1* sequences either with or without normalization to *STE6*, respectively. The *STE6* site was used as a negative Tup1–Cyc8 binding site control. The *STE6* gene promoter is bound by Tup1–Cyc8 in *Mat*α cells, but is free of Tup1–Cyc8 in *Mat*a cells. All strains used in this study were *Mat*a. Primers used are listed in Supplementary Table S2.

### Flocculation assay

Exponentially growing cells were resuspended to an equal cell density in YPD. Equal volumes of cells were aliquoted into a tissue culture plate and agitated by shaking. Five minutes after cessation of agitation, the plates were photographed (30,39). Cells displaying a flocculation phenotype aggregate, and are dispersed in the presence of ethylenediaminetetraacetic acid (EDTA).

## RESULTS

### *FLO1* is the dominant flocculation gene under Tup1–Cyc8 control

**Figure 1.**
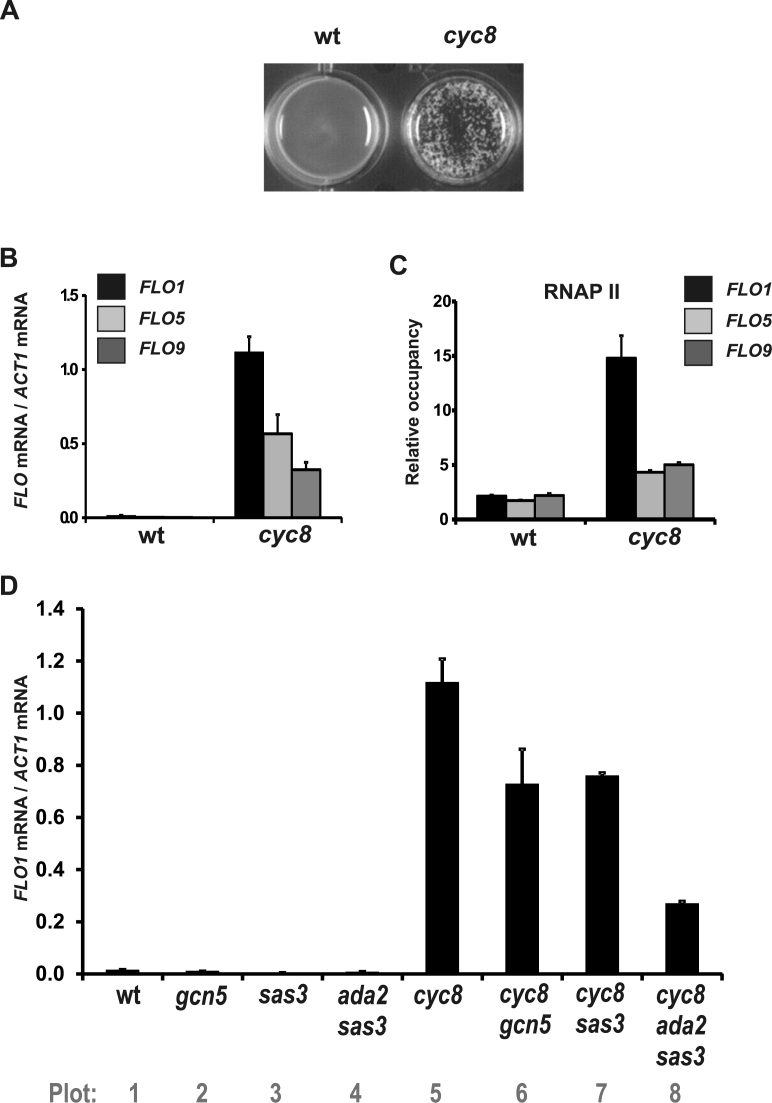
*FLO1* is highly de-repressed in the absence of Tup1–Cyc8. (**A**) Analysis of flocculation in wt and a *cyc8* deletion strain. Flocculation was assayed by photographing yeast strains in liquid culture 5 min after cessation of agitation. (**B**) *FLO1, FLO5* and *FLO9* transcript levels relative to *ACT1* mRNA levels were measured in wt and a *cyc8* deletion strain using RT-qPCR. (**C**) ChIP analysis of RNA polymerase II (RNAP II) occupancy at the *FLO1, FLO5* and *FLO9* gene coding regions in wt and a *cyc8* deletion strain. (**D**) The *ADA2* and *SAS3* genes are redundantly required for *FLO1* de-repression in the absence of Cyc8. *FLO1* transcript levels relative to *ACT1* mRNA levels were measured in wt and strains deleted for the genes indicated. (B–D) The results represent the mean from three to four independent experiments with bars depicting SEM.

The *FLO1* gene encodes a cell wall protein, which mediates cell protection via a flocculation phenotype (5). *FLO1* transcription is repressed by the Tup1–Cyc8 co-repressor complex and is induced by various stresses (10). Initial experiments aimed to determine the contribution of the *FLO* family of genes to the flocculation phenotype observed in the absence of Tup1–Cyc8. In a *cyc8* deletion mutant, which cripples Tup1–Cyc8 occupancy at the *FLO1* promoter, a strong flocculation phenotype was detected, consistent with previous reports (Figure 1A) (26). Analysis of transcripts from the flocculation genes *FLO1, FLO5* and *FLO9* in the *cyc8* mutant by RT-PCR revealed *FLO1* mRNA was the most abundant (Figure 1B). ChIP analysis of RNA polymerase II (RNAP II) occupancy at the various flocculation genes in the absence of *CYC8*, showed RNAP II enrichment was greatest at the *FLO1* gene (Figure 1C). These data suggest Tup1–Cyc8 acts to repress the *FLO1, FLO5* and *FLO9* genes, and that *FLO1* is de-repressed to the greatest extent in the absence of Tup1–Cyc8, consistent with its proposed dominant role in flocculation (7).

### *FLO1* transcription is dependent on Sas3 and Ada2

It has been shown that de-repression of *FLO1* transcription in the absence of Tup1–Cyc8 was accompanied by increased histone acetylation across the promoter (30). However, the HATs responsible for this were unknown. We aimed to identify which HAT was required for the *FLO1* promoter chromatin acetylation and to determine if this acetylation was required for transcription. We therefore examined *FLO1* mRNA levels in wt and *cyc8* mutants additionally deleted for various HATs. If a HAT was required for transcription in the absence of *CYC8*, then its additional deletion in the *cyc8* mutant would reduce the level of *FLO1* de-repression.

We first measured *FLO1* mRNA in wt, *cyc8, gcn5* and *sas3* single and *ada2 sas3* double mutants (Figure 1D). *GCN5* and *ADA2* mutations each cripple the SAGA, ADA and SLIK/SALSA complex HAT activities, whereas a *SAS3* mutation cripples the NuA3 HAT complex (40–44). Since a *gcn5 sas3* double mutant is inviable, an *ada2 sas3* mutant was constructed in order to disable the HAT activities of NuA3 and all Gcn5-containing complexes (45,46). The results showed that *FLO1* was not transcribed in either wt, *gcn5* and *sas3* single mutants or in *ada2 sas3* double mutants, as expected in strains where the Tup1–Cyc8 complex is intact (Figure 1D, plots 1–4). A *cyc8* mutant, on the other hand, displayed a high level of *FLO1* de-repression, as previously reported (Figure 1D, plot 5) (11,26). Upon additional deletion of *gcn5* or *sas3* in the *cyc8* mutant background, the *gcn5 cyc8* and *sas3 cyc8* double mutants displayed a partial reduction in *FLO1* transcript levels compared to the *cyc8* single mutant (Figure 1D, plots 6 and 7). However, an *ada2 sas3 cyc8* triple mutant displayed a significant reduction in *FLO1* mRNA compared to a *cyc8* mutant (Figure 1D, compare plots 8 and 5). This suggests that Gcn5-containing complexes, and the Sas3-containing NuA3 complex, are redundantly required for full *FLO1* de-repression in a *cyc8* mutant background.

### Histone H3 lysine-9 and lysine-14 acetylation at the de-repressed *FLO1* promoter are dependent upon Sas3 and Ada2

The previous results demonstrated that *FLO1* de-repression in the absence of Cyc8 was largely dependent upon *ADA2* and *SAS3*, implicating Gcn5-containing and NuA3 HAT complex activities in the activation of *FLO1* transcription. We next wanted to confirm if the Sas3 and Ada2-dependent HAT activities were responsible for the histone hyperacetylation observed at the de-repressed *FLO1* promoter in the *cyc8* mutant (30). If this were the case, then a decrease in histone acetylation in the *cyc8 ada2 sas3* triple mutant would be predicted, compared to that seen in the *cyc8* single mutant. We therefore examined histone H3 acetylation at lysine-9 (H3K9ac), lysine-14 (H3K14ac) and histone H4 tetra acetylation at lysines-5, -8, -12 and 16 (H4Ac4) across the *FLO1* promoter, by ChIP analysis in single *gcn5* and *sas3* mutants and double *ada2 sas3* mutants in the presence or absence of Cyc8.

**Figure 2.**
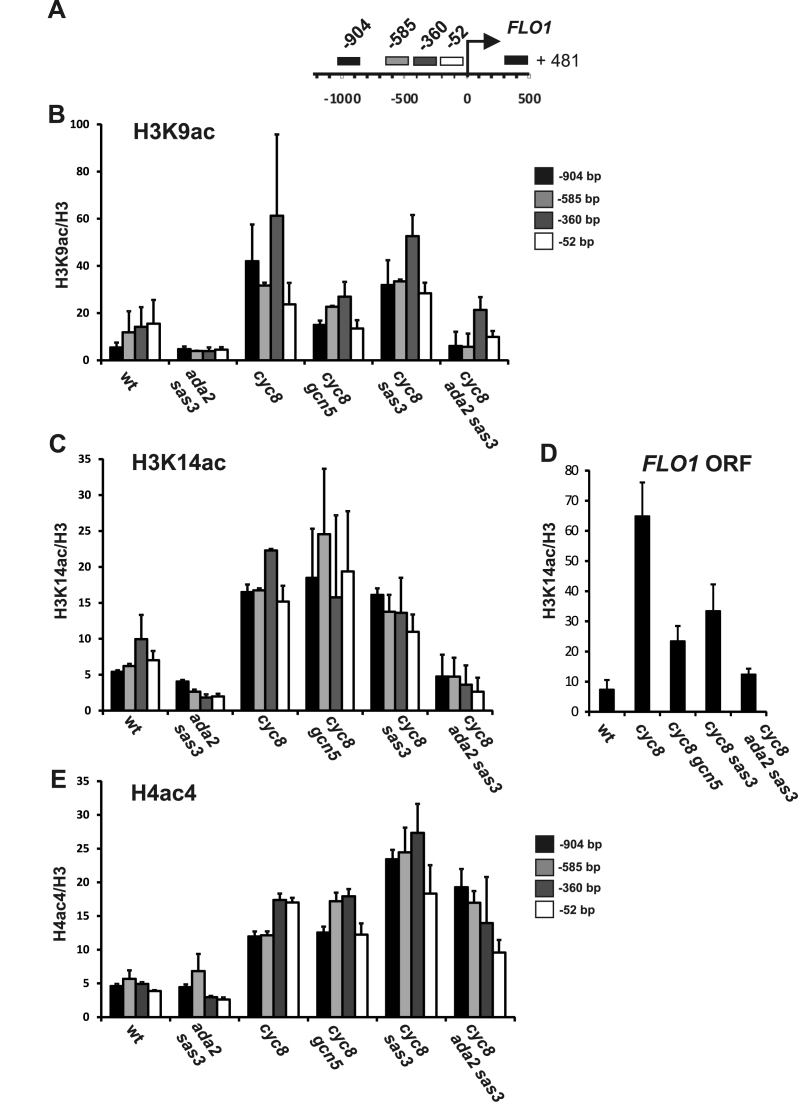
*ADA2* and *SAS3* are required for histone H3K9/K14 acetylation levels at the de-repressed *FLO1* promoter and open reading frame (ORF) in *cyc8* strains. (**A**) Diagram of the amplicons used in ChIP analysis at the *FLO1* promoter and ORF, labeled by the distance (bp) from their midpoints to the *FLO1* translation start site (+1, arrow). Cross-linked chromatin fragments from wild-type (wt) and the mutant strains indicated were immunoprecipitated with antibodies against acetylated histone H3 lysine-9 (**B**, H3K9ac), lysine-14 (**C** and **D**, H3K14ac) and acetylated histone H4 lysines-5, 8, 12 and 16 (**E**, H4ac4). Histone acetylation levels were normalized to *TEL-IV* and are shown relative to histone H3 levels. The results represent the mean from three to four independent experiments with bars depicting SEM. There was no change in acetylation levels compared to wt in the *gcn5* and *sas3* single mutants in which *FLO1* transcription is repressed (Supplementary Figure S1D and data not shown).

Consistent with previously published findings, histone H3 and H4 acetylation was enriched across the *FLO1* promoter region in the *cyc8* mutant where *FLO1* is de-repressed, compared to the low level of acetylation in wt where *FLO1* is repressed (Figure 2B, C and E, compare wt and *cyc8*) (30). When *GCN5* was additionally deleted in the *cyc8* mutant, decreased H3K9ac was detected at the *FLO1* promoter which correlated with the partial decrease in *FLO1* de-repression in this strain compared to the *cyc8* single mutant (Figure 2B, compare *cyc8* and *cyc8 gcn5*). No significant difference in H3K9ac was observed when *SAS3* was deleted in the *cyc8* mutant background compared to the levels in the *cyc8* strain (Figure 2B, compare *cyc8* and *cyc8 sas3*). However, when both *ADA2* and *SAS3* were deleted in addition to *CYC8*, there was a reduction in H3K9ac levels across the *FLO1* promoter compared to the *cyc8* and *cyc8 gcn5* mutants (Figure 2B, compare *cyc8* to *cyc8 ada2 sas3* and *cyc8 gcn5*), which correlated with the low *FLO1* de-repression in the *cyc8 ada2 sas3* strain (Figure 1D).

The high H3K14ac level observed in the *cyc8* mutant was relatively unaffected at promoter sites when either *SAS3* or *GCN5* were additionally deleted, but was significantly reduced in the *cyc8 ada2 sas3* strain (Figure 2C, compare *cyc8* and *cyc8 ada2 sas3*). High H3K14ac levels were also detected in the *FLO1* ORF in the de-repressed *cyc8* strain (Figure 2D, compare wt and *cyc8*). However, these levels were decreased when either *GCN5* or *SAS3* were deleted in addition to *CYC8*, and levels were further decreased when both *ADA2* and *SAS3* were additionally deleted together (Figure 2D, compare *cyc8* and *cyc8 ada2 sas3*). These data suggest Sas3 and Gcn5/Ada2 function redundantly to acetylate H3K14 at the *FLO1* promoter, and work cooperatively for H3K14 acetylation in the ORF.

Although the decrease in H3K9ac levels in the *cyc8 gcn5* and *cyc8 ada2 sas3* mutants compared to *cyc8* were of a similar extent (Figure 2B), only the *sas3 ada2 cyc8* strain showed a significant reduction in *FLO1* de-repression (Figure 1D). This suggests it is the loss of the H3K14ac mark at the *FLO1* promoter and ORF in the absence of Cyc8, Sas3 and Ada2 complexes, which contributes most to the loss of *FLO1* de-repression in the *cyc8 ada2 sas3* mutant. Together, these data suggest that in the absence of Cyc8, Ada2 and Gcn5-containing HAT complexes are responsible for H3K9ac and H3K14ac at the de-repressed *FLO1* promoter, and that the H3K14ac mark contributes most to *FLO1* de-repression. The data also shows that there are high levels of Gcn5 and Sas3-dependent H3K14ac at the de-repressed *FLO1* ORF in the absence of Cyc8.

Histone H4 acetylation (H4ac4) was also increased at the de-repressed *FLO1* promoter in the absence of Cyc8 (Figure 2E, compare wt and *cyc8*). However, the level of this modification was unaffected by the additional loss of Sas3 and Gcn5/Ada2, which is consistent with these enzymes not catalyzing H4ac4 (Figure 2E, compare *cyc8* and *cyc8 sas3 ada2*) (45,47–49).

### The global loss of H3K14ac in *sas3 ada2* mutants does not negatively affect transcription of all genes

**Figure 3.**
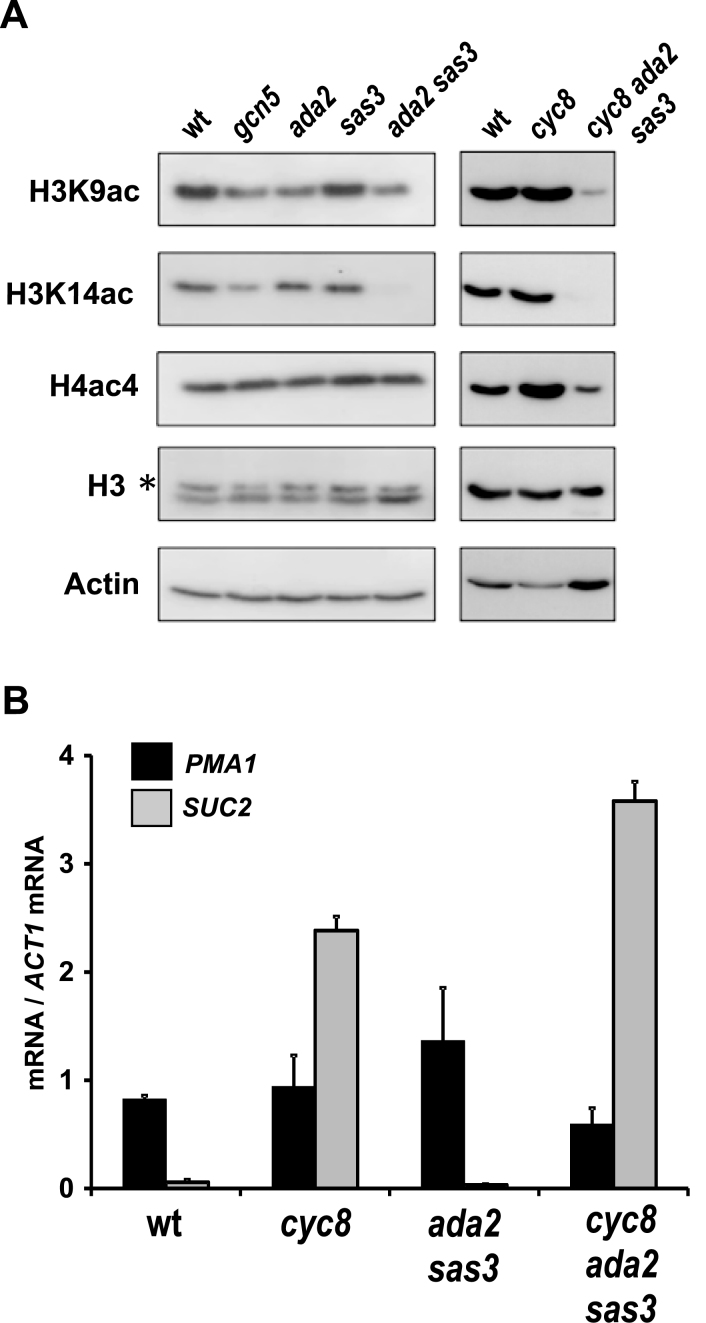
Deletion of *ADA2* and *SAS3* causes the global loss of H3K14ac levels but does not negatively affect transcription of *SUC2* and *PMA1*. (**A**) Whole cell lysates were prepared from wt and the mutant strains indicated. Western blots were probed with antibodies against histone H3, acetylated histone H3 lysine-9 (H3K9ac), acetylated histone H3 lysine-14 (H3K14ac) and acetylated H4 lysines-5, 8, 12 and 16 (H4ac4). Actin served as a loading control in all blots. Variance in H4Ac4 levels did not support a reproducible difference between mutants. All proteins were of the expected sizes. (**B**) *PMA1* and *SUC2* transcript levels relative to *ACT1* mRNA levels were measured in wt and strains deleted for the genes indicated, using RT-qPCR. All cells were grown in YPD. The results represent the mean from three to four independent experiments with bars depicting SEM.

*FLO1* transcription was significantly reduced in *cyc8* mutants deficient for both Ada2 and Sas3 (Figure 1D, compare *cyc8* and *cyc8 ada2 sas3*). This reduction of *FLO1* transcription correlated with reduced levels of H3K9ac at the *FLO1* promoter, and reduced H3K14ac levels at the promoter and ORF, in the *ada2 sas3 cyc8* mutant compared to the *cyc8* mutant (Figure 2B–D, compare *cyc8* and *cyc8 ada2 sas3*). We wanted to confirm if the reduction of acetylation in the HAT mutants was specific to the *FLO1* promoter and ORF, or whether loss of histone acetylation was occurring globally in the mutant strains defective for the two HAT-containing complex activities, as had been reported (45). We therefore measured total H3K9ac, H3K14ac and H4ac4 levels by western blot analysis in whole cell extracts derived from wt, *gcn5, ada2, sas3* and *ada2 sas3* mutants, and also in *cyc8* and *ada2 sas3 cyc8* mutants (Figure 3).

In wt cells, H3K9ac, H3K14ac and H4ac were all detected at significant levels (Figure 3A). Decreased H3K9ac levels were evident in the *gcn5, ada2* and *ada2 sas3* mutants, and there was a reduction of H3K14ac in the *gcn5* mutant compared to wt. Strikingly, H3K14ac levels were undetectable in the *ada2 sas3* strain. Upon the additional loss of Cyc8 in the *ada2 sas3* mutant background (*ada2 sas3 cyc8*), H3K9ac levels were lower than those in wt and H3K14ac was again absent (Figure 3A). There was no loss of histone H3 in any of the mutants, suggesting all decreases in H3 acetylation were lysine-specific. None of the strains tested showed reproducible differences in H4ac levels, consistent with H4 not being a substrate for Gcn5- or Sas3-containing complexes (33,50). These data show that the loss of H3K14ac from the *FLO1* promoter and ORF in the absence of Sas3 and Gcn5 is consistent with the global loss of the modification in this strain (45,50).

However, these data raised the possibility that the reduced *FLO1* de-repression in the *cyc8 ada2 sas3* mutant might be a consequence of a general defect in transcription in this strain due to the global depletion of histone H3K14 acetylation levels. We therefore measured transcript levels of the constitutively active *PMA1* gene, and the glucose repressed *SUC2* gene, in the absence of both Ada2 and Sas3, with and without Cyc8 (Figure 3B).

In the absence of both Ada2 and Sas3, where global H3K14ac levels are abolished, transcription of *PMA1* was not significantly different compared to wt (Figure 3B, black bars, compare wt and *ada2 sas3*). The *cyc8* and *ada2 sas3 cyc8* mutant strains also showed no significant differences in *PMA1* transcription compared to wt. This suggests deletion of both Ada2 and Sas3, and the resultant global loss of H3K14ac, either in the absence or presence of Cyc8, has no significant negative effect on *PMA1* transcription.

We also analyzed transcription from *SUC2*, which is a glucose repressed gene under Tup1–Cyc8 control (Figure 3B, gray bars) (25,51,52). As expected, after growth in glucose-containing media, wt cells showed no *SUC2* transcription due to Tup1–Cyc8 mediated glucose repression. *SUC2* transcription was also absent in an *ada2 sas3* mutant, suggesting the Tup1–Cyc8 complex and glucose repression remains functional in this strain. Conversely, glucose-grown *cyc8* mutants showed a high level of *SUC2* de-repression due to the absence of Tup1–Cyc8 causing relief from glucose repression (Figure 3B, compare wt and *cyc8*). Importantly, in the *cyc8* mutant additionally deleted for both *ADA2* and *SAS3, SUC2* transcription was also de-repressed to a similar, or even higher level, to that seen in the *cyc8* mutant (Figure 3B, compare *cyc8* and *cyc8 ada2 sas3*). This suggests that despite the global loss of H3K14ac due to the absence of Ada2 and Sas3, there is no impact on transcription at other genes in the *ada2 sas3 cyc8* mutant. Furthermore, the data suggests that not all Tup1–Cyc8 regulated genes require H3K14ac for de-repression since, unlike transcription of *FLO1, SUC2* de-repression was unaffected by the loss of Ada2 and Sas3 in the *cyc8* deletion background.

### Histone H3 lysine-14 acetylation plays the major role in *FLO1* de-repression

**Figure 4.**
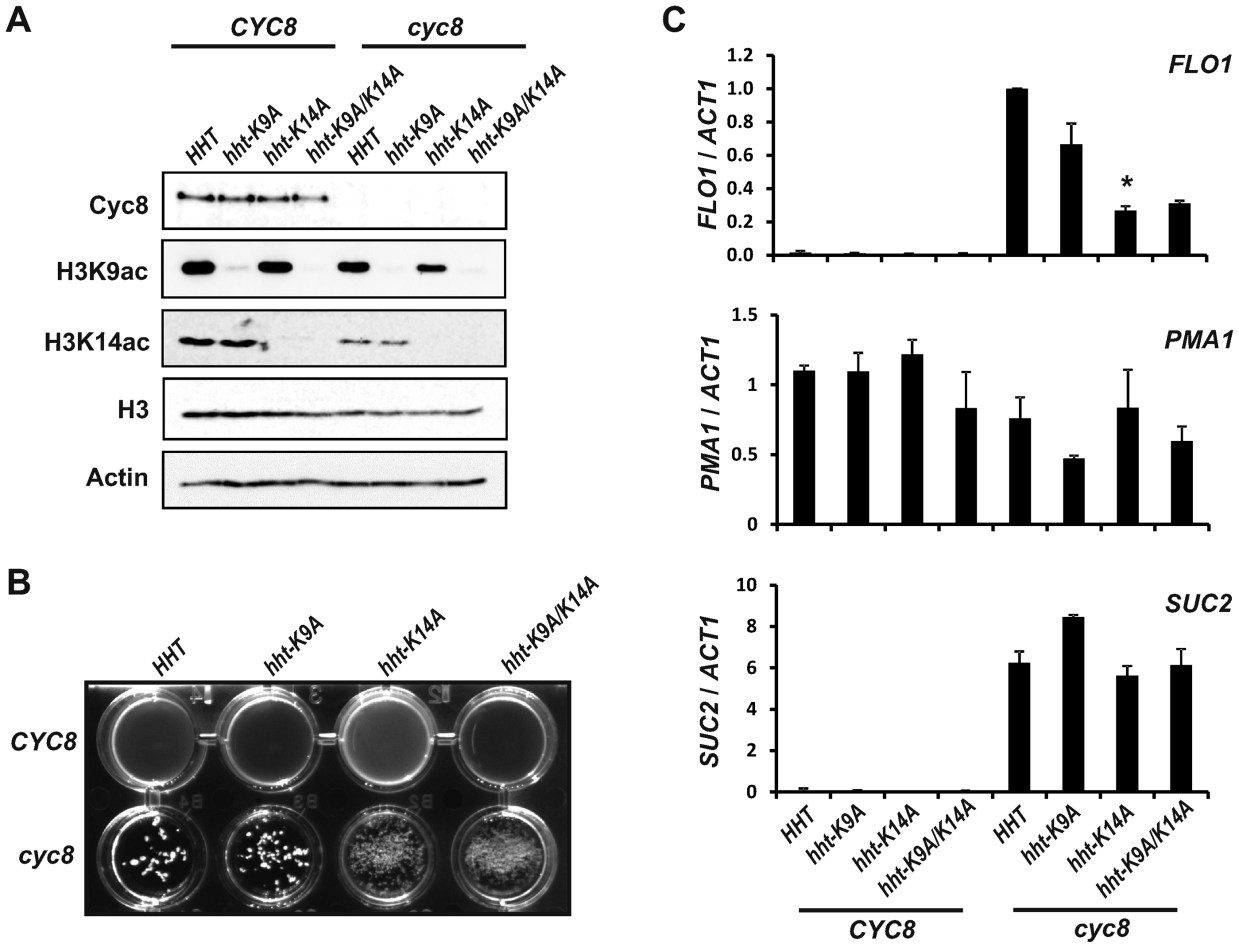
Histone H3 lysine-14 acetylation plays the major role in *FLO1* de-repression. (**A**) Western blot analysis to confirm construction of site-specific histone acetylation-deficient mutants either with or without a *CYC8* gene deletion. Strains were constructed in which the histone H3 gene (*HHT*) was altered to yield mutant versions of H3 containing lysine to alanine substitutions at amino acid residues -9 (*hht-K9A*) and -14 (*hht-K14A*), either singly, or combined (*hht-K9A/K14A*), and with and without a *CYC8* gene deletion. All proteins were of the expected sizes. (**B**) Analysis of flocculation in wt (*HHT CYC8*) and strains deficient for the histone acetylation sites indicated, either in the presence or absence of a *CYC8* gene deletion. Flocculation was visualized as described for Figure 1A. (**C**) *FLO1, PMA1* and *SUC2* transcript levels relative to *ACT1* mRNA levels were measured in the strains indicated using RT-qPCR. All cells were grown in YPD. The results represent the mean from three independent experiments with bars depicting SEM. The asterisk indicates a statistically significant difference between the *hht1-K14A cyc8* strain and both *cyc8 HHT1* and *hht1-K9A cyc8* strains as determined by the Student's *t*-test (*P* < 0.005).

We next wanted to establish whether the positive role of *SAS3* and *GCN5*-dependent complexes in *FLO1* de-repression was occurring directly via their acetylation of H3K9 and H3K14 residues, and not via indirect effects due to their possible acetylation of other histone residues or non-histone proteins. We therefore constructed yeast strains expressing mutant versions of histone H3 containing lysine to alanine (K to A) substitutions at residues 9 and 14, either singly or combined, and examined *FLO1* transcription in these strains in the presence and absence of *CYC8*. The mutant versions of histone H3 are the sole source of histone H3 in these strains, and cannot be acetylated at these sites (Figure 4A).

If H3K9ac and H3K14ac contributed directly to *FLO1* de-repression in the absence of Cyc8, then H3K9ac and H3K14ac deficient mutants additionally deleted for *CYC8* should show less *FLO1* de-repression than the *cyc8* mutant in the wt H3 background (*HHT*). No flocculation was apparent in any of the yeast strains expressing the mutant histones when Cyc8 was present (Figure 4B, upper wells). There was also no significant difference in flocculation and *FLO1* de-repression in the *cyc8* mutant whether in the H3K9ac-deficient histone mutant (*cyc8 hht-K9A*) or wt histone background (*cyc8 HHT*) (Figure 4B and C, *FLO1*). By contrast, compared to the *cyc8* mutant, flocculation and *FLO1* transcription was decreased when *CYC8* was deleted in the histone H3K14ac-deficient background (Figure 4B and C, *FLO1*; compare *cyc8 HHT* and *cyc8 hht-K14A*). Flocculation and *FLO1* de-repression were similarly decreased when *CYC8* was deleted in the histone mutant background deficient for both H3K9 and K14 acetylation (Figure 4B and C, *FLO1*; compare *cyc8 HHT* and *cyc8 hht-K9A/K14A*). Together, this confirms H3K14ac makes the dominant contribution to *FLO1* de-repression in the absence of *cyc8*. Importantly, compared to *FLO1* transcription levels in the *cyc8* mutant, the decrease in *FLO1* transcription in the *cyc8 hht-K14A* strain was similar to the decrease in the *cyc8 ada2 sas3* mutant (compare Figures 1D and 4C, *FLO1*).

Together, these data suggest that the role of Sas3 and Ada2 upon *FLO1* transcription in the absence of Cyc8 is predominantly occurring directly via acetylation of lysine 14 of histone H3, and not indirectly via acetylation of alternative histone sites or other non-histone proteins. Furthermore, the data suggests the requirement of H3K14ac for *FLO1* de-repression is gene specific since the global loss of H3K14 acetylation in the histone acetylation deficient mutant does not negatively affect transcription of the constitutively expressed *PMA1* gene, or de-repression of the glucose repressed *SUC2* gene (Figure 4C, *PMA1* and *SUC2*; compare *cyc8 HHT* and *cyc8 hht-K14A*).

### Gcn5 and Sas3 predominantly occupy the de-repressed *FLO1* promoter and ORF, respectively

**Figure 5.**
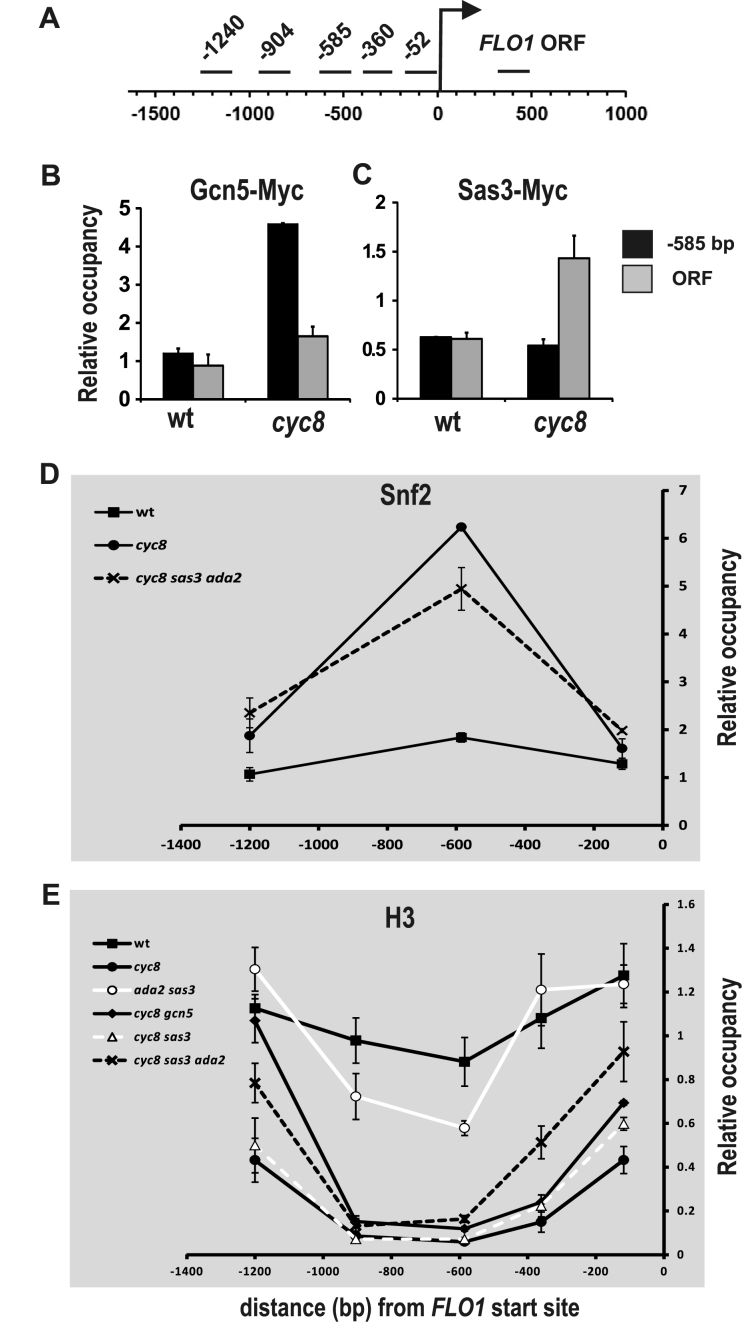
Gcn5 and Sas3 predominantly occupy the *FLO1* promoter and ORF, respectively, in the absence of Tup1–Cyc8. (**A**) Diagram of the amplicons used in ChIP analysis at the *FLO1* promoter and ORF were as described in Figure 2A. (**B**) ChIP analysis of Gcn5-Myc in wt and *cyc8* strains expressing Myc-tagged Gcn5. (**C**) ChIP analysis of Sas3-Myc in wt and *cyc8* strains expressing Myc-tagged Sas3. (**D**) Snf2 occupancy at the *FLO1* promoter in the absence of Tup1–Cyc8 is not dependent on *ADA2* and *SAS3*. ChIP analysis of the Snf2 sub unit of the Swi–Snf complex at the *FLO1* promoter in the strains indicated. (B–D) Gcn5-Myc, Sas3-Myc and Snf2 levels were normalized to *TEL-IV*. (**E**) Histone H3 occupancy is reduced over the *FLO1* promoter region in all *cyc8* mutant backgrounds regardless of whether *FLO1* is de-repressed or not. Histone H3 ChIP at the *FLO1* promoter in wt and the mutant strains indicated was normalized to the *INT-V* region. (B–E) The results represent the mean from three to four independent experiments with bars depicting SEM.

Our results suggest that the HAT activities of Sas3 and Gcn5-containing complexes are required for histone H3K14 acetylation at the *FLO1* promoter and ORF in the absence of Cyc8, and that this contributes to *FLO1* de-repression. To test if Gcn5 and Sas3 were acting directly at *FLO1*, we used ChIP to determine if these proteins occupied the de-repressed *FLO1* promoter and ORF when Cyc8 was absent. In the wt strain where Tup1–Cyc8 is present, *FLO1* transcription is repressed and *FLO1* promoter chromatin shows little histone acetylation, we could not detect enrichment of either Myc-tagged Gcn5 or Sas3 (Figure 5B and C). Conversely, in the absence of Cyc8, when *FLO1* promoter and ORF chromatin is hyperacetylated and transcription is de-repressed, enrichment of Gcn5 was confirmed at a region −585 bp proximal to the *FLO1* transcription start site, and was also detected at low levels in the ORF (Figure 5B). However, Myc-tagged Sas3 was only detectable at the *FLO1* ORF in the de-repressed *cyc8* strain (Figure 5C). These data reveal that Gcn5 is recruited to the same site previously occupied by Tup1–Cyc8 at the de-repressed *FLO1* promoter and show it is also present at low levels in the *FLO1* ORF, where it is enriched together with Sas3.

### *FLO1* chromatin acetylation, and not nucleosome eviction, correlates with *FLO1* transcription

The de-repression of *FLO1* transcription in the absence of Tup1–Cyc8 is accompanied by extensive nucleosome eviction across the *FLO1* promoter and upstream region (22,26,30). Both *FLO1* transcription and this chromatin remodeling have been attributed to Swi–Snf, since Snf2 has been shown to occupy the de-repressed *FLO1* promoter and histone eviction and transcription are abolished in a *cyc8 snf2* mutant (26,30,53). The data presented so far suggest that Ada2 and Sas3-dependent histone H3K14 acetylation at the de-repressed *FLO1* promoter and ORF contributes to *FLO1* de-repression. One model for the role of the Ada2 and Sas3-dependent histone acetylation in *FLO1* de-repression would be that it aids Swi–Snf binding at the *FLO1* promoter to catalyze nucleosome rearrangement and eviction, thus making the *FLO1* promoter permissive for transcription (54–59). This model would predict that in the *cyc8* mutant additionally deleted for Gcn5 and Sas3, in which *FLO1* de-repression and promoter acetylation are significantly reduced, Swi–Snf occupancy and histone eviction would also be reduced.

We therefore examined Snf2 occupancy at the *FLO1* promoter in the presence and absence of Cyc8 either with or without both Sas3 and Ada2(Gcn5). We confirmed Snf2 was recruited to the de-repressed *FLO1* promoter region in the absence of Cyc8 (Figure 5D, compare wt and *cyc8*) (30). However, in the *cyc8 sas3 ada2* mutant where *FLO1* transcription is impaired, we found that Snf2 was present at levels similar to that in the fully de-repressed *cyc8* mutant (Figure 5D, compare *cyc8 ada2 sas3* and *cyc8*). Thus, Sas3 and Ada2-dependent *FLO1* promoter acetylation is not required for Swi–Snf recruitment.

We next examined histone H3 occupancy at the *FLO1* promoter in the presence and absence of Cyc8 either with or without Sas3 and Ada2 (Figure 5E). In wt cells, where *FLO1* transcription is repressed, uniform H3 occupancy levels were confirmed across the *FLO1* promoter region (Figure 5E, wt). Similarly, in the *sas3 ada2* mutant in which Tup1–Cyc8 is present and *FLO1* is repressed, histone H3 levels were also generally as high as in wt cells, although a slight decrease in levels was detected at the −585 and −905 regions (Figure 5E, compare wt and *ada2 sas3*). Conversely, in the *cyc8* strain, where *FLO1* is highly de-repressed, extensive histone depletion across the *FLO1* promoter and 1 kb upstream region was apparent, as has been reported (Figure 5E, compare wt and *cyc8*) (22,26). In the *cyc8* mutant additionally deleted for either of the HATs Sas3 or Gcn5, and where *FLO1* de-repression is similar to that in *cyc8* mutant, histone levels were also similarly reduced as in the *cyc8* mutant. Thus, for all these strains, low *FLO1* transcription correlates with high promoter histone occupancy, and *vice-versa*.

However, in the *cyc8* mutant additionally deleted for both Sas3 and Ada2 (*cyc8 sas3 ada2*) and where *FLO1* promoter acetylation and transcription are significantly reduced compared to the *cyc8* strain, extensive histone eviction was still evident (Figure 5E, compare *cyc8* and *cyc8 ada2 sas3*). Thus, in the absence of Tup1–Cyc8, Sas3 and Ada2, the proposed Swi–Snf-dependent histone eviction at the *FLO1* promoter still occurs, but *FLO1* transcription remains largely repressed. These data show that Swi–Snf recruitment and histone eviction activity at the *FLO1* promoter occur independently of histone H3K9/14 acetylation. These data also suggest it is not histone eviction alone that dictates whether the *FLO1* promoter is permissive for transcription, but the Sas3 and Ada2(Gcn5)-dependent acetylation of the residual chromatin template is also required for *FLO1* transcription.

### Chromatin remodeling at the *SUC2* promoter occurs concomitant with transcription after conditional depletion of Cyc8 from the nucleus.

**Figure 6.**
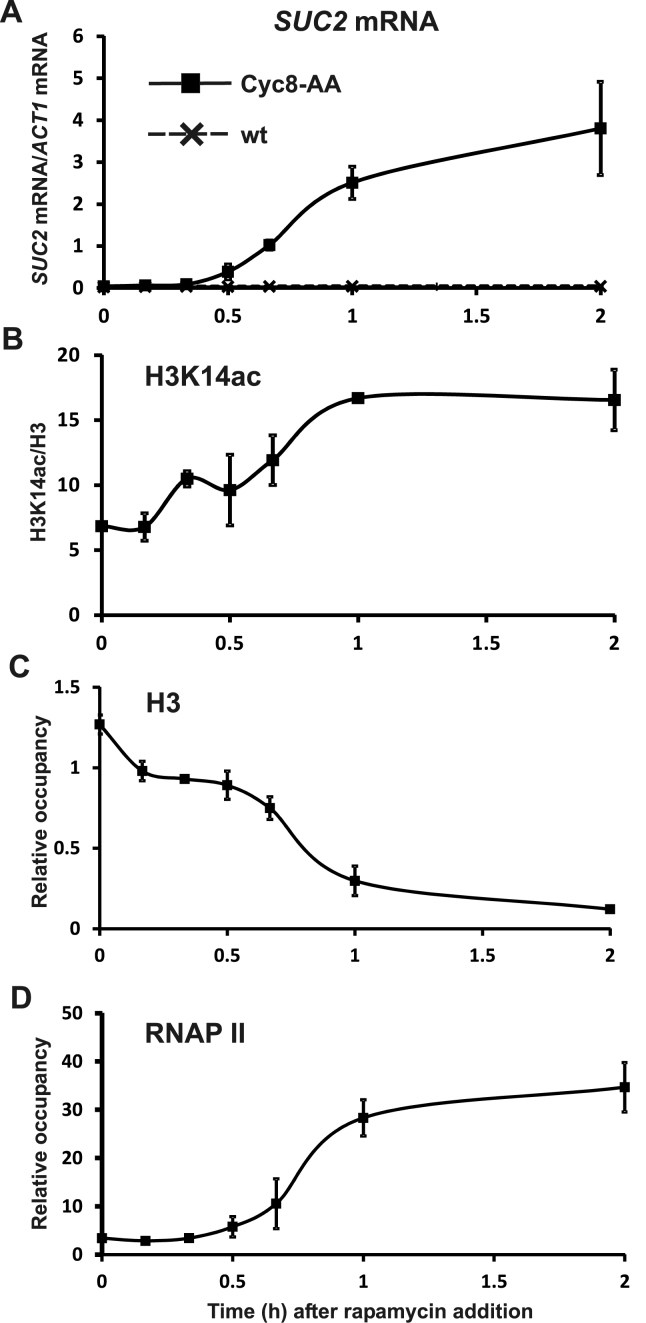
Conditional depletion of Cyc8 via the anchor-away technique results in robust *SUC2* de-repression and concomitant promoter chromatin remodeling. (**A**) *SUC2* transcript levels relative to *ACT1* were measured in the control (wt) and Cyc8 anchor-away strain (Cyc8-AA) at the times indicated (h) after rapamycin treatment. Time-course ChIP analysis measuring the occupancy of (**B**) histone H3 lysine-14 acetylation, (**C**) histone H3 and (**D**) RNA polymerase II (RNAP II) at the *SUC2* promoter in the Cyc8 anchor-away strain (Cyc8-AA) after rapamycin addition. H3 and RNAP II occupancy were normalized to levels at the ORF-free regions, *INT-V* and *TEL-IV*, respectively. H3K14ac levels were normalized to *TEL-IV* and are shown relative to histone H3 levels. (A–D) The results represent the mean from three to four independent experiments with bars depicting SEM.

To gain more insight into the mechanism of *FLO1* de-repression we used the anchor-away technique to conditionally deplete Cyc8 from the nucleus and then monitored *FLO1* de-repression and the occupancy of RNA polymerase II (RNAP II), H3, Snf2 and histone H3K14 acetylation (H3K14ac) at the *FLO1* promoter over time (37). As a control for the technique, we first examined events at another Tup1–Cyc8 regulated gene, *SUC2* (Figure 6). *SUC2* encodes the enzyme invertase, which is required for sucrose metabolism and is subject to glucose repression via Tup1–Cyc8 (51,52). Under conditions of low glucose, or in the absence of Tup1–Cyc8, *SUC2* is de-repressed (19,60–62).

We first measured *SUC2* mRNA accumulation in a strain expressing FRB-tagged Cyc8 (Cyc8-FRB) after depletion of Cyc8-FRB via the addition of rapamycin. Consistent with previously published results, there was significant *SUC2* mRNA accumulation within 60 min of rapamycin addition (Figure 6A) (24). ChIP analysis revealed that *SUC2* promoter histone acetylation also peaked at 60 min post-rapamycin addition (Figure 6B) concurrent with the maximum loss of histone H3 (Figure 6C), and increased occupancy of RNAP II (Figure 6D). This confirmed the anchor-away technique worked, and showed that following Cyc8-FRB depletion, histone acetylation and eviction at the *SUC2* promoter correlated with *SUC2* transcription (24).

### Histone H3 eviction at the *FLO1* promoter coincides with Cyc8 depletion.

**Figure 7.**
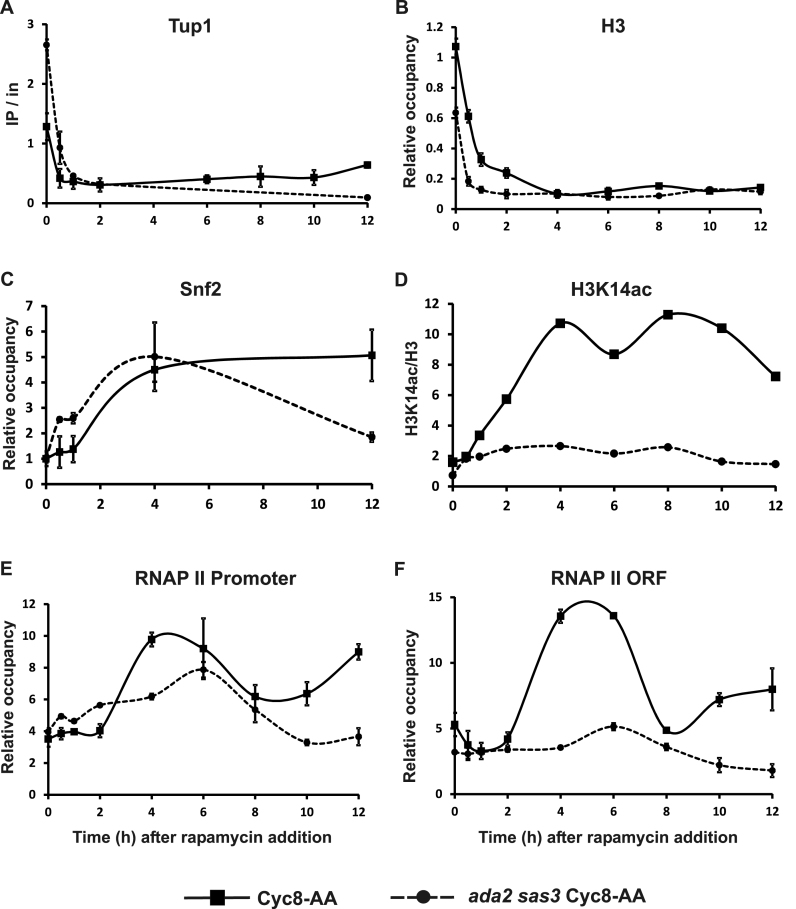
De-repression of *FLO1* following the depletion of Cyc8 via anchor-away involves the biphasic recruitment of RNAP II to the *FLO1* promoter and ORF, and is dependent upon Ada2 and Sas3. Time-course ChIP analysis measuring the occupancy of (**A**) Tup1, (**B**) histone H3, (**C**) Snf2, (**D**) H3K14ac at the *FLO1* promoter and (**E** and **F**) RNAP II at the *FLO1* promoter and ORF respectively, in the Cyc8-anchor-away (Cyc8-AA) and *ada2 sas3* Cyc8-anchor-away (*ada2 sas3* Cyc8-AA) strains at the times indicated (h) after rapamycin addition. Tup1 occupancy was measured relative to input DNA. H3, Snf2 and RNAP II occupancies were normalized to *INT-V, ACT1* and *TEL-IV* regions, respectively. H3K14ac levels were normalized to *TEL-IV* and are shown relative to histone H3 levels. The results represent the mean from three to four independent experiments with bars depicting SEM except for (D), which is the average of two independent experiments (Supplementary Figure S3).

We next examined the occupancy of Tup1, H3, H3K14ac, Snf2 and RNAP II at the *FLO1* promoter over time after Cyc8 removal via anchor-away (Figure 7). Since Tup1–Cyc8 occupancy at the *FLO1* promoter is dependent on the Cyc8 sub unit, we measured Tup1 levels at the *FLO1* promoter by ChIP as an indicator of the presence of the Tup1–Cyc8 complex in the Cyc8 anchor-away strain (30). Following rapamycin addition, Tup1 was rapidly lost from the *FLO1* promoter, showing significant depletion by 30 min after treatment (Figure 7A). This rate of loss of Tup1 (and Cyc8) from *FLO1* was similar to the rate of Tup1 depletion from *SUC2* and other target sites following its anchor-away (24).

In the absence of Tup1–Cyc8, Swi–Snf is required for *FLO1* transcription and the accompanying histone eviction which occurs across the *FLO1* promoter and upstream region (26,53). Using ChIP analysis, we measured the occupancy of the Snf2 catalytic subunit of the Swi–Snf remodeling complex, and histone H3, at the *FLO1* promoter following Cyc8 depletion (Figure 7B and C). The data showed that significant histone H3 eviction was evident by 30 min after rapamycin addition (Figure 7B), which mirrored the rate of loss of Tup1 from the *FLO1* promoter (compare Figure 7A and B). However, maximum H3 eviction was not achieved until 4 h, which coincided with maximum Snf2 recruitment at the *FLO1* promoter (Figure 7B and C). Snf2 occupancy then persisted until the 12 h time point together with the continued absence of H3 from the promoter throughout this time period (Figure 7B and C). Since Swi–Snf is essential for *FLO1* promoter remodeling and transcription (26), the apparent delay in Snf2 occupancy compared to the significant early histone H3 eviction is unexplained, and requires further investigation. We note that the lag in detection of DNA-bound Snf2 by ChIP does not exclude a prior association of the large Swi–Snf complex that is out with DNA cross-linking distance. In addition, the requirement for Swi–Snf does not rule out that it may remodel and evict in concert with a histone chaperone, as was observed at the *HO, PHO5* and *PHO8* promoters (63–65). Together, these data show that following Cyc8 depletion, rapid histone H3 eviction occurs at the *FLO1* promoter which is accompanied by Snf2 recruitment, with maximal Snf2 occupancy and histone eviction coinciding 4 h after rapamycin addition.

### Biphasic histone H3K14 acetylation and RNAP II recruitment occurs at the *FLO1* promoter after Cyc8 depletion

The data presented so far suggests the Sas3 and Gcn5-containing HAT complex dependent histone H3K14 acetylation of the residual *FLO1* promoter chromatin template after nucleosome eviction is critically required for *FLO1* transcription. We therefore examined H3K14ac and RNAP II occupancy over time at the *FLO1* promoter and ORF following Cyc8 anchor-away (Figure 7D–F). Two reproducible peaks of H3K14ac occupancy occurred at the *FLO1* promoter at 4 and 8 h post rapamycin addition (Figure 7D and Supplementary Figure S3). Interestingly, the first peak of H3K14ac coincided with a peak in RNAP II recruitment to the *FLO1* promoter and ORF, whereas the second peak of H3K14ac preceded a second peak of RNAP II at 12 h post rapamycin addition (Figure 7D–F). Thus, there was a reproducible biphasic pattern of H3K14ac at the *FLO1* promoter which coincided with a similar and more striking biphasic pattern of RNAP II recruitment to the *FLO1* promoter and ORF. RNAP II occupancy at the *SUC2, PMA1* and *BAP2* genes in the rapamycin treated cells did not show significant fluctuations in RNAP II occupancy (Supplementary Figure S4). Therefore, the biphasic RNAP II occupancy profile at *FLO1* after rapamycin addition is not a general response of RNAP II under these conditions.

### *FLO1* mRNA accumulates gradually after conditional depletion of Cyc8

**Figure 8.**
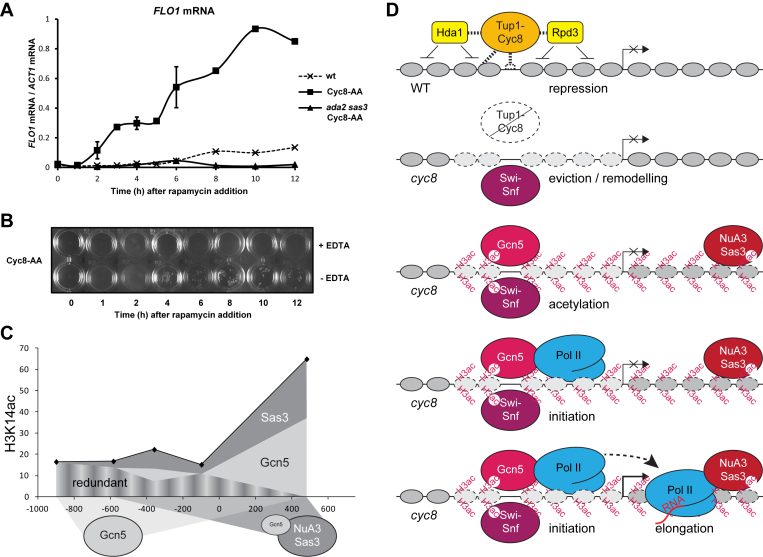
The conditional depletion of Cyc8 results in gradual *FLO1* gene de-repression and flocculation. (**A**) *FLO1* transcript levels relative to *ACT1* were measured by RT-qPCR in the control (wt), Cyc8-anchor-away (Cyc8-AA) and *ada2 sas3* Cyc8-anchor-away (*ada2 sas3* Cyc8-AA) strains at the times indicated (h) after rapamycin treatment. The results represent the mean from three independent experiments with bars depicting SEM. (**B**) Flocculation in the Cyc8 anchor-away (Cyc8-AA) strain at the times indicated (h) after rapamycin treatment. Flocculation was assayed as described in Figure 1A. (**C**) Relative contributions of Gcn5 and Sas3 to the H3K14ac profile in de-repressed *cyc8* strains, as calculated from the H3K14ac reduction in *cyc8 gcn5* and *cyc8 sas3* mutants (Figure 2C and D). Low levels in *cyc8 ada2 sas3* mutants combined with low transcription suggest Gcn5 and Sas3 are the primary HATs (Figures 1D, 2C and D). Over the promoter, a modest decrease in acetylation in the absence of either HAT indicates their activities are redundant. In the coding region, substantial effects on histone acetylation indicate HAT activities are largely cumulative. Figure 5B indicates these HATs largely occupy either the promoter (Gcn5) or ORF (Sas3), suggesting they can control histone acetylation at a distance. (**D**) Diagram showing different stages of *FLO1* gene de-repression. The initiation-elongation transition is not observed in the absence of histone acetylation. Refer to text in the ‘Discussion’ section for details.

The kinetic analysis of events at *FLO1* following Cyc8 depletion shows that Tup1 loss and histone eviction occur together relatively rapidly, and peak at 1 h after treatment, which was similar to the time-scale of events at *SUC2* (compare Figures 6 and 7). However, unlike at *SUC2*, maximum RNAP II recruitment did not occur until 4 h after treatment, followed by a second peak of RNAP II recruitment at 12 h (Figure 7E and F). We therefore examined if *FLO1* transcription and flocculation also followed this pattern of RNAP II recruitment. Surprisingly, *FLO1* mRNA accumulation did not mirror the biphasic RNAP II profile and accumulated slowly over time (Figure 8A). Indeed, by 2 h after rapamycin addition, *FLO1* mRNA was only just detectable and did not reach a peak until 10 h after rapamycin treatment. Importantly, the maximum level of *FLO1* mRNA attained following Cyc8 anchor-away was similar to that detected in the *cyc8* deletion strain (compare Figures 8A and 1B). The flocculation phenotype also developed gradually after Cyc8 depletion (Figure 8B), and correlated with *FLO1* mRNA accumulation. These data suggest both *FLO1* transcript levels and flocculation increase gradually after Cyc8 depletion, in contrast to the more rapid de-repression observed for *SUC2*.

Together, these data show that following rapamycin addition, Cyc8–Tup1 is rapidly depleted from the *FLO1* promoter which undergoes similarly rapid histone H3 eviction, and Snf2 recruitment. In addition, the acetylation of the remodeled chromatin template occurs in two waves, which precede two peaks of RNAP II recruitment to the *FLO1* promoter and gene coding region. Importantly, whereas H3 has been significantly evicted from the *FLO1* promoter by 1 h after Cyc8 depletion, the acetylation and recruitment of RNAP II occurs later, first peaking at 4 h post rapamycin addition, and again after 8 and 12 h, respectively. These data reveal that histone H3 eviction is not the sole driver for *FLO1* transcription, but show the acetylation status of the residual chromatin after histone eviction correlates with, and is potentially required for, RNAP II transcription at *FLO1*. These data are consistent with the results from the initial deletion mutant analysis.

### In the absence of Sas3 and Ada2, entry of RNAP II into elongation at the *FLO1* ORF is impaired

The data suggest that Sas3 and Ada2-dependent histone H3K14 acetylation at the *FLO1* promoter is required for *FLO1* transcription. In order to confirm the role of Sas3 and Ada2 at the *FLO1* promoter during de-repression, we repeated the Cyc8 anchor-away experiment in a strain additionally deleted for both *SAS3* and *ADA2. FLO1* transcription and the occupancy of Tup1, H3, H3K14ac, Snf2 and RNAP II at the *FLO1* promoter were analyzed over time (Figure 7).

Consistent with the steady-state deletion mutant results, ChIP analysis showed histone H3K14 acetylation was severely reduced in the Sas3 and Ada2 mutant Cyc8 anchor-away strain (*ada2 sas3* Cyc8-AA) after rapamycin addition (Figure 7D). Tup1 was depleted at a similar rate to the wt anchor-away strain (Cyc8-AA) in this double mutant background, suggesting the anchor-away of Cyc8 from the *FLO1* promoter was unaffected by the absence of Sas3 and Ada2 and the loss of histone H3 K9/14 acetylation (Figure 7A). Histone H3 eviction at the *FLO1* promoter also proceeded as rapidly as in wt following rapamycin addition (Figure 7B). Similarly, recruitment of Snf2 to the *FLO1* promoter was unaffected by the *SAS3* and *ADA2* deletion (Figure 7C). However, Snf2 occupancy was lower at the 12 h time point in the double mutant compared to wt, suggesting Snf2 is less stable at the *FLO1* promoter in the absence of Sas3 and Ada2 during the later times following Cyc8 depletion.

Importantly, despite the significant and rapid loss of H3 from the *FLO1* promoter following Cyc8 depletion in the Sas3 and Ada2 mutant background, no *FLO1* mRNA was detected in this strain (Figure 8A, compare *ada2 sas3* Cyc8-AA and Cyc8-AA). This confirms Sas3 and Ada2 are required for *FLO1* de-repression in the absence of Cyc8. These data also reveal that Snf2 recruitment and histone eviction occur independently of histone acetylation, and that promoter histone eviction is not in itself sufficient for *FLO1* transcription.

Surprisingly, RNAP II occupancy was detected at the *FLO1* promoter in the Sas3 and Ada2 double mutant following rapamycin addition, although it was recruited at a slower rate, and to a lower level, as compared to the wt anchor-away strain (Figure 7E). Furthermore, the peak of RNAP II occupancy in the HAT mutant which occurred at 6 h dropped to pre-rapamycin addition levels by 10 h after treatment and a second peak of RNAP II, as seen in wt, was not detected within the time frame of the study. Most strikingly however, no RNAP II could be detected in the *FLO1* ORF in the *SAS3* and *ADA2* double mutant at any time following rapamycin addition (Figure 7F). Together, this suggests that in the absence of Sas3, Ada2 and H3K14ac, RNAP II is recruited to the histone-depleted *FLO1* promoter, but fails to enter into productive elongation and is lost from the promoter. Thus, the data is consistent with Sas3 and Ada2-dependent HAT activities at *FLO1* being required for the transition of RNAP II at the *FLO1* promoter from initiation to elongation, and for the second peak of RNAP II enrichment.

Analysis of RNAP II at the *SUC2* promoter following Cyc8 anchor-away showed that its recruitment and function was not diminished by the absence of Sas3 and Ada2 (Supplementary Figure S5). The absolute requirement for H3K14ac at *FLO1* in determining RNAP II transitions and stability, although not a universal response, reveals a distinct new role for Sas3 and Gcn5-containing complex-dependent H3K14ac in the regulation of transcription activation.

## DISCUSSION

The *FLO1* gene is under the antagonistic control of the Tup1–Cyc8 co-repressor and the Swi–Snf co-activator complexes and is a good model system in which to investigate chromatin-mediated regulation of transcription. Although the HDACs involved in Tup1–Cyc8 regulation of *FLO1* repression have been identified and characterized (30), the HATs responsible for the acetylation that accompanies *FLO1* de-repression had not been investigated for their role in this process.

Our genetic analysis identified Sas3 and Ada2 as required for *FLO1* transcription (Figure 1D). Since an *ADA2* deletion cripples Gcn5 HAT activity in the context of the ADA, SAGA and SLIK complexes, these data implicate the Sas3-containing NuA3 complex and Gcn5-containing complexes as the partially redundant HAT activities in the de-repression of *FLO1* transcription (45).

We showed that the Sas3 and Gcn5-containing HAT complexes are essential for transcription, predominantly via their acetylation of histone H3 lysine 14 at the de-repressed *FLO1* promoter and ORF (Figures 2C, D and 4C). The data further indicates that Sas3 and Gcn5 function redundantly to acetylate H3K14 at the de-repressed promoter, and work cooperatively to acetylate H3K14 in the ORF (Figures 2C, D and 8C). Gcn5 was strongly recruited to the de-repressed *FLO1* promoter, and was also detectable at low levels in the ORF, whereas Sas3 was only detectable in the ORF (Figure 5B and C). These results are consistent with a dominant role for Gcn5-mediated histone acetylation at the de-repressed *FLO1* promoter with contribution at a distance from Sas3, in addition to the local role for Sas3 and also Gcn5 for acetylation in the ORF (Figure 8C). The wide histone H3 acetylation range of these HATs may be explained by their self-stabilising chromatin contacts and the 3D chromatin architecture at the transcription initiation site (66).

Previously published data showed the chromatin remodeling complex Swi–Snf was required for *FLO1* de-repression in the absence of Tup1–Cyc8 via its catalysis of nucleosome rearrangement and eviction across the *FLO1* promoter and upstream region (26,30,53). Evidence suggests that promoter histone acetylation can facilitate the recruitment and stability of chromatin remodeling complexes such as Swi–Snf which act as co-activators at target genes (31,56,58). Indeed, the bromodomain of the Snf2 sub unit of Swi–Snf has been proposed to stabilize this complex at acetylated chromatin regions (55,67). By contrast, Swi–Snf occupancy in the *cyc8* mutant additionally deleted for *SAS3* and *ADA2*, did not appear to correlate with the reduced histone H3K9/K14 acetylation and *FLO1* transcription in this strain.

Snf2 occupancy and extensive histone eviction were detected at the highly acetylated de-repressed *FLO1* promoter in the absence of Cyc8, as has been previously reported (Figure 5D and E) (22,30). However, in the *cyc8 ada2 sas3* mutant, in which *FLO1* promoter H3K9/14 acetylation and transcription are significantly decreased, Snf2 occupancy and histone eviction levels at the *FLO1* promoter were the same as in the *cyc8* mutant (Figure 5D and E). Thus, Swi–Snf recruitment and histone eviction at the promoter in the absence of Cyc8 are not dependent upon Sas3 and Ada2-dependent histone H3K9 and K14 acetylation. Taken together, these data suggested that *FLO1* de-repression is not solely dependent upon histone eviction, and that Sas3 and Gcn5-containing complex-dependent acetylation of the chromatin template after histone eviction, was also a key requirement for *FLO1* de-repression in the absence of Cyc8.

To gain further insight into the role of Sas3 and Ada2-dependent histone H3 acetylation upon *FLO1* de-repression we used the anchor-away technique to conditionally and rapidly deplete Cyc8 from the nucleus (24,37). Since the loss of Cyc8 abolishes Tup1–Cyc8 occupancy at *FLO1*, this technique allowed us to monitor the time-course of events at the *FLO1* promoter observed during steady state gene de-repression (30). At the glucose repressed *SUC2* gene, which is also under Tup1–Cyc8 control (52,60), anchor-away of Cyc8 resulted in significant transcription of *SUC2* by 1 h post-rapamycin addition, consistent with previously published results (24). This de-repression was accompanied by concurrent histone loss, histone H3K14 acetylation and RNAP II recruitment at the *SUC2* promoter (Figure 6). However, *FLO1* mRNA was only detectable 2 h after rapamycin addition, and did not peak until after 10 h (Figure 8A). The slow *FLO1* mRNA accumulation was not due to slow depletion of Cyc8–Tup1 from the *FLO1* promoter, since significant loss of Tup1 was detected after 30 min (Figure 7A). Almost concurrent with the rapid Tup1 depletion, histone H3 levels also dramatically decreased at the *FLO1* promoter (Figure 7B). Consistent with the *FLO1* promoter chromatin remodeling and transcription following Cyc8 depletion, Snf2 occupancy was enriched at the promoter with maximum occupancy at 4 h (Figure 7C).

Coincident histone H3K14 acetylation of the *FLO1* promoter was observed with maximum levels also peaking at 4 h post-rapamycin addition. Surprisingly however, the histone H3K14 acetylation profile at *FLO1* revealed a second peak which occurred at 8 h post rapamycin addition (Figure 7D). Furthermore, RNAP II occupancy at the *FLO1* promoter and ORF mirrored the H3K14 acetylation pattern, whereby two phases of RNAP II enrichment were also evident; one coinciding with maximum H3K14 acetylation levels at 4 h, and the other appearing after the second acetylation peak, at 12 h post rapamycin addition (Figure 7E and F). The outcome of the biphasic recruitment of RNAP II to the *FLO1* promoter is a gradual accumulation of *FLO1* mRNA (Figure 8A). Analysis of RNAP II occupancy at the Tup1–Cyc8 regulated *SUC2* gene, *BAP2* and the constitutively expressed *PMA1* gene in the anchor-away strain following rapamycin addition, did not show this biphasic pattern of RNAP II occupancy (Supplementary Figure S4).

When the Cyc8 depletion via anchor-away was repeated in a strain additionally deleted for *SAS3* and *ADA2*, both H3K14ac and *FLO1* de-repression were abolished (Figures 7D and 8A). Importantly however, Snf2 recruitment to the *FLO1* promoter and histone eviction were unaffected (Figure 7B and C). This suggests histone H3K14 acetylation is not required for Snf2 recruitment or its activity, and that histone eviction is not in itself sufficient to enable *FLO1* transcription, consistent with the results from the steady state deletion mutant analysis.

Surprisingly, despite the absence of *FLO1* mRNA in the double HAT deficient Cyc8 anchor-away strain, recruitment of RNAP II at the *FLO1* promoter was evident, although it was delayed, reached slightly lower levels, and the second peak of RNAP II enrichment was not detected within this time frame. However, RNAP II occupancy at the *FLO1* ORF was largely abolished in this strain throughout the entire Cyc8 depletion time-course (Figure 7E and F). Together, these data suggest that although Ada2 and Sas3 play a role in RNAP II recruitment to the *FLO1* promoter in the absence of Cyc8, their major role is to enable the subsequent transition from transcription initiation to elongation.

The role for Ada2 and Sas3 in *FLO1* transcription elongation could be due to either, (i) their acetylation of the depleted *FLO1* promoter chromatin or other proteins is required for RNAP II release into the ORF, (ii) their acetylation of histones within the *FLO1* ORF is required to enable entry of RNAP II into the gene coding region, or (iii) their concerted promoter and ORF acetylation activities are both required to ensure a successful transition from initiation to elongation occurs. In support of a role for acetylation within the *FLO1* ORF being required for RNAP II elongation is our data showing Sas3, and to a lesser extent Gcn5, as well as Sas3 and Ada2-dependent H3K14 acetylation can all be detected in the de-repressed *FLO1* ORF in the absence of *CYC8* (Figures 2D and 5B and C).

Recent studies have also shown the Gcn5-containing SAGA complex is active at almost all RNAP II transcribed genes in yeast and humans (68). Moreover, there is evidence that Gcn5-dependent acetylation within the ORF of inducible genes in *S. cerevisiae* and *S. pombe* promotes histone eviction which enables RNAP II elongation to occur (69,70). Furthermore, Gcn5 within the SAGA complex has been shown to cooperate with the transcription-coupled NuA4 HAT complex which catalyzes H4ac4 within coding regions to mediate histone eviction to stimulate transcription elongation (71). Taken together, we propose that the major role for Ada2 and Sas3 in the regulation of *FLO1* transcription is via their acetylation of *FLO1* ORF histones at H3 lysine-14 to enable RNAP II elongation to occur, perhaps by promoting histone eviction.

It is interesting to note that although the absence of Sas3 and Ada2 had a negative impact on *FLO1* de-repression, the *SUC2* gene, which is similarly repressed by Tup1-Ssn6, was not subject to this H3K14ac-dependent regulation. Evidence suggests that *gcn5* mutants displayed decreased RNAP II processivity and mRNA production at long (4.5 kb), but not short (1.5 kb) genes that were driven by the *GAL1* promoter (69,72). Thus it may be the relatively long length of the *FLO1* gene (4.6 kb) that makes it more dependent upon H3K14 acetylation for transcription elongation than the shorter (1.6 kb) *SUC2* gene.

We cannot exclude the possibility that the *ada2* mutation may disrupt the DUB module of the SAGA complex necessary for H2B deubiquitylation during transcription elongation, which could cause an elongation defect (73,74). However, this is unlikely since *FLO1* de-repression was similarly reduced in the *cyc8 ada2 sas3* mutant and in the histone H3K14ac-deficient H3K9A/K14A *cyc8* strain, in which the SAGA complex is intact (Figures 1D and 4C).

A role in elongation for the NuA3 complex carrying the Sas3 HAT activity is also becoming clearer. Based on subunit composition, two sub complexes have been distinguished: NuA3a is thought to acetylate H3K14 over the promoter, whereas NuA3b interacts with H3K36 methylation in gene coding regions (75). Recent genome wide data reported that Sas3 is located preferentially within the promoter proximal coding regions of target genes, supporting its possible involvement in the transcriptional elongation process (76). This is consistent with our ORF based location for Sas3, while we also observed Sas3 activity at a distance contributing to H3K14ac over the promoter (Figure 8C). The emerging roles in elongation for NuA3 complexes and Gcn5-containing complexes can explain their post transcription initiation involvement in the rate-limiting control of RNAP II elongation. Moreover the cooperation we see between Sas3 and Ada2 for elongation at *FLO1* is consistent with their frequent co-localization within the coding regions of other highly active genes (77).

### A model for coordinated but separate events of chromatin remodeling and histone acetylation

DNA access in chromatin is proposed to be facilitated by the coordinated action of chromatin-modifying enzymes and ATP-dependent chromatin remodelers. A current model states that Swi–Snf is recruited to regulatory regions via interaction with site specific DNA binding factors, while subsequent stabilization of its chromatin association by interactions between bromodomains and acetylated histones leads to preferential remodeling of these nucleosomes (55,56,78). The view that histone acetylation acts by slowing the Swi–Snf off-rate can unify observation of variations in the order of recruitment of remodeling and modifying complexes at different promoters (79). Genome wide analysis indicates that groups of genes have different dependencies of Swi–Snf occupancy based on either histone H3 acetylation or activators (80).

Interestingly, on the de-repressed *FLO1* promoter, Swi–Snf binding and remodeling activity is independent of H3 acetylation, but does not seem subject to a high off-rate (Figure 5D and E). However, H4ac4 is also significantly increased over the *cyc8* de-repressed *FLO1* gene promoter where it could provide a substrate for Swi–Snf stabilization on the inactive *cyc8 ada2 sas3* template (Figures 2E and 5D). Indeed, the Snf2 bromodomain has similar binding preferences for SAGA-acetylated histones and H3K9/14 acetylated peptides, as for NuA4-acetylated histones and H4K5/8/12/16 acetylated peptides. Moreover, both H3 and H4 acetylation were also shown to enhance early Swi–Snf binding at the *SUC2* gene (81,82). Absence of Gcn5-mediated Snf2 protein acetylation would also remove a competitive internal substrate for its bromodomain, allowing it to bind lower levels of acetylated histones (83). Nevertheless, lack of H3K9/K14 acetylation in the inactive *cyc8 ada2 sas3* mutant might explain the slow off-rate eventually observed for Swi–Snf compared to its stable binding in *cyc8* cells, despite initially faster Swi–Snf recruitment and eviction rates (Figure 7B and C).

We compared the results at *FLO1* with events at *SUC2*, another Tup1–Cyc8 repressed gene also known to require Swi–Snf dependent promoter remodeling in addition to histone acetylation upon induction. At the *SUC2* promoter, acetylation of H3 by recruitment of the SAGA complex upon induction is not dependent on Swi–Snf remodeling and occurs alongside high basal acetylation of H3 (84,85). However, following induction, Cyc8–Tup1 is retained or increased on the de-repressed *SUC2* promoter where it is proposed to convert into a coactivator and shifts location to facilitate recruitment of SAGA (84,85). Thus, glucose de-repression of *SUC2* transcription requires rapid Gcn5 and Swi–Snf recruitment, in addition to the persistence of Tup1–Cyc8 occupancy (81).

By contrast, after depletion of Cyc8 by anchor-away, we observe that *SUC2* transcription is independent of Gcn5 and Sas3, and in fact does not require H3K9/K14 acetylatable residues (Figures 3B and 4C). This situation may be similar to the *GAL1* gene, which in *tup1* or *cyc8* deletion mutants was enabled for transcription in the absence of Gcn5 (85). Thus, in common with *FLO1, SUC2* initiation in the absence of Tup1–Cyc8 does not require histone H3 acetylation through HAT recruitment. The gene-repressive effect of Tup1–Cyc8 is due to its association with multiple HDACs (18,30). Therefore, the requirement for Gcn5 and Sas3 in transcription initiation could be to counteract these antagonistic HDAC activities or to open Tup1–Cyc8 organized chromatin (30). Gcn5 and Sas3 have been similarly proposed to antagonize ISWI remodeling activity (86). Although not Tup1–Cyc8 regulated, *PMA1* is a housekeeping gene that is regulated by metabolic state and has a Mediator requirement classifying it as a SAGA-dependent gene (87,88). While in our study it is in a constitutively expressing non repressed state, *PMA1* expression is also only minimally affected by mutations in HATs or H3 acetylatable residues (Figures 3B and 4C).

A time-course analysis of *SUC2* induction after glucose de-repression reported biphasic transcription patterns (81). Interestingly, we did not observe this activation behavior upon Cyc8 anchor-away depletion at *SUC2* (Figure 6). The timeline suggests that the initial surge of transcription did not occur, which may therefore be dependent on Cyc8, or on low glucose signalling events that permit rapid switching on of *SUC2* gene activity. However, in our anchor-away conditions we did observe biphasic transcription patterns at *FLO1*. We confirm the different nature of these two *FLO1* phases, as the second H3K14ac wave appears to result in lower or delayed RNAP II activity. The cell metabolic explanations proposed for biphasic *SUC2* activity do not match the slow activation timing at *FLO1;* instead population density, nutrient sensing and cell signalling would appear to form the basis of this observation.

## CONCLUSION

In summary, we have identified that the Sas3 and Gcn5-containing HAT complexes are required for *FLO1* de-repression in the absence of Cyc8 and that this role is mediated via histone H3K14 acetylation at the *FLO1* promoter and ORF (Figure 8D). Following the rapid depletion of Cyc8 from the nucleus, Snf2 recruitment, histone eviction, histone acetylation and RNAP II recruitment precede the gradual accumulation of *FLO1* mRNA. Our data show histone eviction at the de-repressed *FLO1* promoter is independent of Sas3 and Ada2-mediated histone H3K9 and K14 acetylation, and that promoter histone eviction is essential, but not sufficient for, *FLO1* de-repression. The data reveals RNAP II is recruited to the *FLO1* promoter and ORF in two waves following Cyc8 depletion, concomitant with a similar histone acetylation profile. In the absence of Sas3 and Ada2-dependent H3K9/14 acetylation, only the first peak of enrichment of RNAP II was detected within this time frame at the promoter and no occupancy can be detected in the ORF. This suggests Sas3 and Ada2-dependent H3K14 acetylation is not required for RNAP II recruitment to the de-repressed *FLO1* promoter, but is required for the transition from transcription initiation to elongation (Figure 8D). We propose the histone acetylation-dependent regulation of RNAP II elongation and gradual *FLO1* mRNA accumulation ensures the slow expression of the flocculation phenotype occurs within a cell population so that cells expressing the Flo1 protein do not lose their competitive advantage (5).

A new insight gained from this study is that histone H3 acetylation promotes and stabilizes transcription initiation events without being integral or essential to the events leading to RNAP II recruitment at the promoter. Instead, we confirm histone H3 acetylation is necessary to counteract repressors and associated HDAC activities that commonly repress gene promoters. Further distinction of the requirements for histone acetylation in these mechanisms, made possible by the slower kinetics in our study, revealed a separate RNAP II initiation-elongation transition requirement for Gcn5 and Sas3 activities in the gene 5΄ coding region that is novel and essential for *FLO1* transcription. This was not required at *SUC2*, a short gene subject to rapid on/off sensing, or at *PMA1*, during constitutive expression. At *FLO1* it is proposed to rate-limit the expression of cell–cell adhesive properties that require control at population level.

## Supplementary Material

Supplementary Data
