# Supplementary material for: Sas3 and Ada2(Gcn5)-dependent histone H3 acetylation is required for transcription elongation at the de-repressed FLO1 gene
Source: Nucleic Acids Res. 2017 Jan 23;45(8):4413–30. doi: 10.1093/nar/gkx028 (PMC5416777; doi:10.1093/nar/gkx028)
Supplement: Supplementary Data [file gkx028_Supp.pdf]

Church et al., Table S1. Yeast strains used in this study.

Strain	Genotype	Description	Source
BY4741	*Mata his3Δ1 leu2Δ0 met15Δ0 ura3Δ0*	Wt, Fig. 1-3&5	(Brachmann et al., 1998)
YMC29	*Mata his3Δ1 leu2Δ0 met15Δ0 ura3Δ0 cyc8::LEU2*	*cyc8*, Fig. 1-3&5	This study
YAFTCD1	*Mata his3Δ1 leu2Δ0 met15Δ0 ura3Δ0 gcn5::KAN*	*gcn5*, Fig. 1	Euroscarf
YAFYBL052c	*Mata his3Δ1 leu2Δ0 met15Δ0 ura3Δ0 sas3::KAN*	*sas3*, Fig. 1	Euroscarf
YMC22	*Mata his3Δ1 leu2Δ0 met15Δ0 ura3Δ0 sas3::kanMX4 ada2::URA3*	*ada2 sas3*	This study
YMC16	*Mata his3Δ1 leu2Δ0 met15Δ0 ura3Δ0 gcn5::KANMX4 cyc8::URA3*	*cyc8 gcn5*	This study
YMC20	*Mata his3Δ1 leu2Δ0 met15Δ0 ura3Δ0 sas3::KANMX4 cyc8::URA3*	*cyc8 sas3*	This study
YMC27	*Mata his3Δ1 leu2Δ0 met15Δ0 ura3Δ0 sas3::KANMX4 ada2::URA3 cyc8::LEU2*	*cyc8 ada2 sas3*	This study
YAF1	DY20D (W303A: *MATa; ura3-52; trp1Δ2; leu2-3_112; his3-11; ade2-1; can1-100, hhf2-hht2::NAT*, *hta1-htb1::HPH*, *hht1-hhf1::KAN*, *hta2-htb2::NAT*). [pRS314; *COPY1 TRT1*]	*HHT1 CYC8*, Fig. 4	Mary Ann Osley
YAF5	DY20D (W303A: *MATa; ura3-52; trp1Δ2; leu2-3_112; his3-11; ade2-1; can1-100, hhf2-hht2::NAT*, *hta1-htb1::HPH*, *hht1-hhf1::KAN*, *hta2-htb2::NAT*) [pRS314; *copy1-K9A TRT1*]	*hht1-K9A*, Fig. 4	Mary Ann Osley
YAF7	DY20D (W303A: *MATa; ura3-52; trp1Δ2; leu2-3_112; his3-11; ade2-1; can1-100, hhf2-hht2::NAT*, *hta1-htb1::HPH*, *hht1-hhf1::KAN*, *hta2-htb2::NAT*).[pRS314; *copy1-K14A TRT1*]	*hht1-K14A*, Fig. 4	Mary Ann Osley
YAF17	DY20D (W303A: *MATa; ura3-52; trp1Δ2; leu2-3_112; his3-11; ade2-1; can1-100, hhf2-hht2::NAT*, *hta1-htb1::HPH*, *hht1-hhf1::KAN*, *hta2-htb2::NAT*).[pRS314; *copy1-K9A/K14A TRT1*]	*hht1-K9/K14A*, Fig. 4	Mary Ann Osley
YAF1DF15	YAF1, *cyc8::URA3*	*HHT1 cyc8*, Fig. 4	This study
YAF5DF26	YAF5, *cyc8::URA3*	*hht1-K9A cyc8*, Fig. 4	This study
YAF7DF11	YAF7, *cyc8::URA3*	*hht1-K14A cyc8*, Fig. 4	This study
YAF17DF1	YAF17, *cyc8::URA3*	*hht1-K9A/K14A cyc8*, Fig. 4	This study
YMC14	*Mata his3Δ1 leu2Δ0 met15Δ0 ura3Δ0 GCN5-*9myc*::HPH1*	Wt GCN5-Myc, Fig. 5B	This study
YMC15	*Mat a his3Δ1 leu2Δ0 met15Δ0 ura3Δ0 GCN5-*9Myc*::HPH1 ssn6::URA3*	*cyc8* GCN5-Myc, Fig. 5B	This study
YMC32	*Mat a his3Δ1 leu2Δ0 met15Δ0 ura3Δ0 SAS3-9Myc::HPH1*	Wt Sas3-Myc, Fig. 5B	This study
YMC66	*Mat a his3Δ1 leu2Δ0 met15Δ0 ura3Δ0 SAS3-9Myc::HPH1 cyc8::URA3*	*cyc8* Sas3-Myc, Fig. 5B	This study
HHY221	*Mata tor1-1 fpr1::loxP-LEU2-loxP RPL13A-2×FKBP12::loxP*	Wt, Fig. 6A	(Haruki et al., 2008)
YMC30	*Mata tor1-1 fpr1::loxP-LEU2-loxP RPL13A-2×FKBP12::loxP CYC8-FRB::HIS3*	Cyc8-AA, Fig. 6-8	This study
YMC33	*Mata tor1-1 fpr1::loxP-LEU2-loxP RPL13A-2×FKBP12::loxP CYC8-FRB::HIS3 ada2::URA3 sas3::KANMX4*	*ada2 sas3* Cyc8-AA, Fig. 7-8	This study

Table S2. Oligonucleotides used in qPCR.

Name:	Sequence (5'-3'):	Description:	Distance relative to ATG:
**TEL VI-R 121 F**	CGTGTGTAGTGATCCGAACTCAGT	Control region	N/A
**TEL VI-R 121 R**	GACCCAGTCCTCATTTCCATCAATAG		
**Int-V-F**	TAAGAGGTGATGGTGATAGGCGT	Control region	N/A
**Int-V-R**	CCCTCGGGTCAAACACTACAC		
**IPSTE6-F**	GATATGGCTGAACTATCTCCCG	Control region	-60
**IPSTE6-R**	GCTTGTTCTTTGTTTCCTAGTGG		
**PMA1 ORF-F**	GAAAAAGAATCTTTAGTCGTTAAGTTCGTT	Control region	+322
**PMA1 ORF-R**	AATTGGACCGACGAAAAACATAA		
**ACT1 ORF-F**	GAGGTTGCTGCTTTGGTTATTGA	Control region	+318
**ACT1 ORF-R**	ACCGGCTTTACACATACCAGAAC		
**FLO1RT-F**	TACCACCACAGACGGGTTCT	*FLO1* transcription/ ORF ChIP	+481
**FLO1RT-R**	CAACAGTTGAACGCGGTTGC		
**FLO5RT5'-F2**	GGATGGAAGTCTCCCTGACA	*FLO5* transcription	+635
**FLO5RT5'-R2**	GGAAACGGCATTGGAGTAAA		
**FLO9RT5'-F**	TCGTCACATTGCTGGGATTA	*FLO9* transcription	+105
**FLO9RT5'-R**	TGCTGCATTCGAATATGTGG		
**SUC2RT486-F**	AGCTGCCAACTCCACTCAAT	*SUC2* transcription	+486
**SUC2RT486-R**	ATTTGGCAGCCGTCATAATC		
**IPFLO1-F**	AAAGGAACATATTTCACTCTTGCTC	*FLO1* ChIP	-52
**IPFLO1-R**	TCTGTTTACTGGTGACAAGAATTAAAA		
**IPFLO2-F**	TGTGGAACCTTCTACAGTACTTCGG	*FLO1* ChIP	-360
**IPFLO2-R**	TTTGAGTGCCTTTCAACAATTTCAGACTT		
**IPFLO3-F**	GCTTCCAGTATGCTTTCACG	*FLO1* ChIP	-585
**IPFLO3-R**	GCCTACGTATTCTCCGTCAC		
**IPFLO4-F**	AGTCTCATTACCTAAACGCCAG	*FLO1* ChIP	-904
**IPFLO4-R**	CTGAAACTGGCTAGCATAACAC		
**IPFLO5-F**	TTGAATGGCACTAGTCGATCG	*FLO1* ChIP	-1240
**IPFLO5-R**	TTAAACTTACGGCATCTTGAACATT		
**NUC4-F (TATA)**	TGGAAGAAAGATTTGACGACTTT	*SUC2* ChIP	-168
**NUC4-R (TATA)**	TGTTTCTTTTCAGGAGGAAGGA		
**BAP2 5' F**	ATCCGGGAGTGACAAACTTATAC	"Constitutive" gene	+132
**BAP2 5' R**	ACTCAACGCCATCCTCTAAATC		

Table S3. Antibodies used in Western immunoblotting.

Protein	Concentration	Source
β-actin	1:3,000	Abcam (ab8224)
Myc	1:5,000	Millipore (05-724)
Cyc8	1:500	Santa Cruz (sc-11953)
H3	1:5,000	Active Motif (39163)
H3K9ac	1:3,000	Millipore (07-352)
H3K14ac	1:2,500	Millipore (07-353)
H4ac4	1:6,000	Millipore (06-866)
FRB	1:2000	Enzo (ALX-215-065-1)

All antibodies were diluted in 5 % skimmed milk in Tris-buffered saline with 0.05% Tween 20 (TBST). Blots were developed with ECL Western Blotting Substrate (Pierce).

Table S4. Antibodies and conditions used for chromatin immunoprecipitation (ChIP).

Antibody	Amount of antibody (µl)	Protein A or G	Number of washes	Source of antibody
Tup1	1.5	A	2	J. Reese
RNAP II	4.5	A/G mix	2	Covance (MMS-126R)
Myc	2.5	G	2	Millipore (05-724)
H3	4	A	1	Active Motif (39163)
H3K9ac	2.5	G	2	Millipore (07-352)
H3K14ac	2.5	G	2	Millipore (07-353)
H4ac4	3	G	2	Millipore (06-866)
Snf2-N	2.5	G	1	J. Reese

Antibodies were added to cell lysates and incubated with rotation at 4°C overnight. Histone H3 was pre-bound to 30 µl protein A Dynabeads (Life Technologies) prior to overnight incubation with cell lysates at 4°C with rotation. The anti-Snf2 and anti-Tup1 antibodies were generous gifts from J. Reese.

With the exception of histone H3, the chromatin-antibody-bead complexes were collected by the addition of 30 µl of the appropriate Dynabead or Dynabeads mix and incubation for 2 hours at 4°C.

All chromatin-antibody-bead complexes were washed in 1 ml FA lysis buffer for 5 minutes, followed by either one or two washes (as indicated in Table S4) in 1 ml ChIP wash buffer #1 (50 mM HEPES [pH 7.5], 0.5 M NaCl, 1 mM EDTA, 1 % Triton X-100, 0.1 % Sodium deoxycholate), either one or two washes (as indicated in Table S4) in 1 ml ChIP wash buffer #2 (10 mM Tris-Cl [pH 8.0], 0.25 M LiCl, 1 mM EDTA, 0.5 % NP-40, 0.5 % Sodium deoxycholate) and a single wash in 1 ml TE (pH 7.5). Beads were then resuspended in 250 µl ChIP elution buffer (25 mM Tris Cl [pH 7.5], 5 mM EDTA, 0.5 % SDS) and the chromatin was eluted after the sequential incubation at 65°C for 20 minutes, rotation for 10 minutes at

room temperature and centrifugation at 16,363 rcf for 1 minute. The resultant supernatant was protease treated and the cross-links were reversed. Input (in) and immunoprecipitated DNA (IP) were purified using a QiaQuick PCR purification kit (Qiagen) according to manufacturer’s instructions.

DNAs were analysed in triplicate by real-time quantitative PCR (qPCR) using a SYBR Green Master Mix (ABI) and ABI Step-One Plus PCR machine.

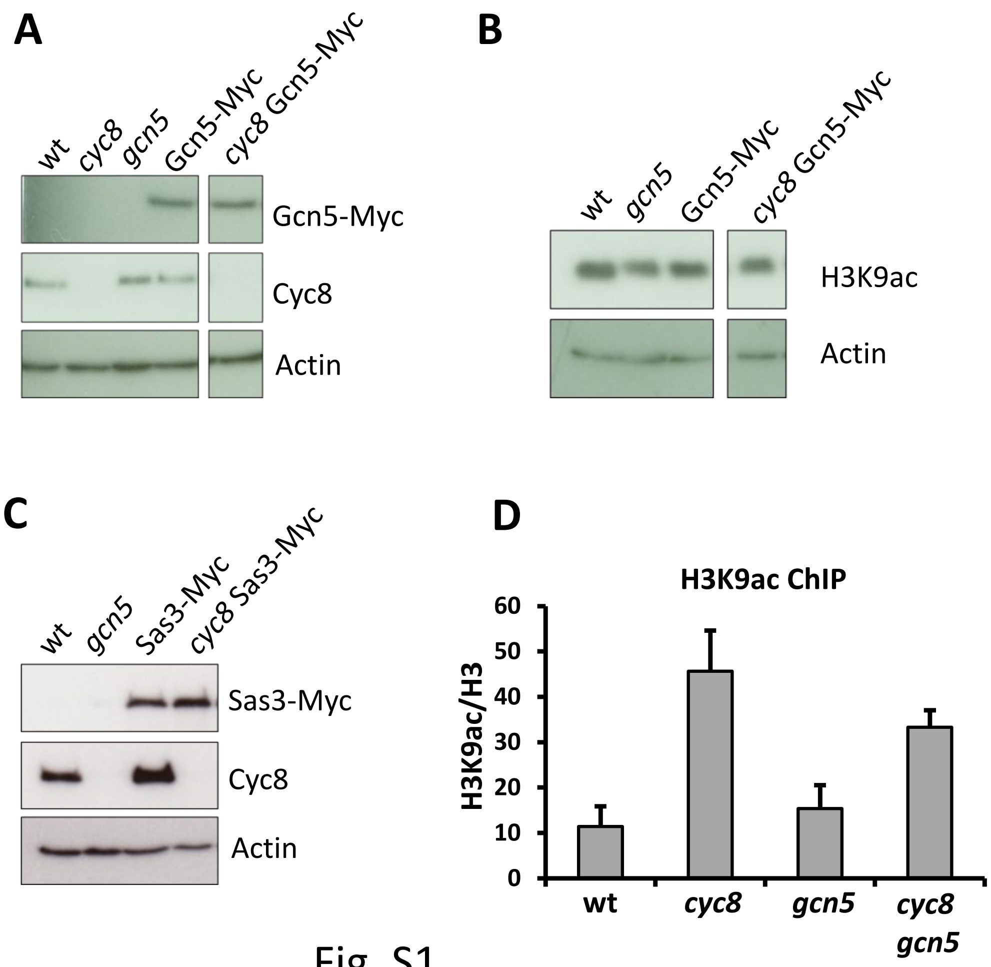
A
wt
cyc8
gcn5
Gcn5-Myc
cyc8 Gcn5-Myc
Gcn5-Myc
Cyc8
Actin
B
wt
gcn5
Gcn5-Myc
cyc8 Gcn5-Myc
H3K9ac
Actin
C
wt
gcn5
Sas3-Myc
cyc8 Sas3-Myc
Sas3-Myc
Cyc8
Actin
D
H3K9ac ChIP
H3K9ac/H3
60
50
40
30
20
10
0
wt
cyc8
gcn5
cyc8 gcn5

Fig. S1

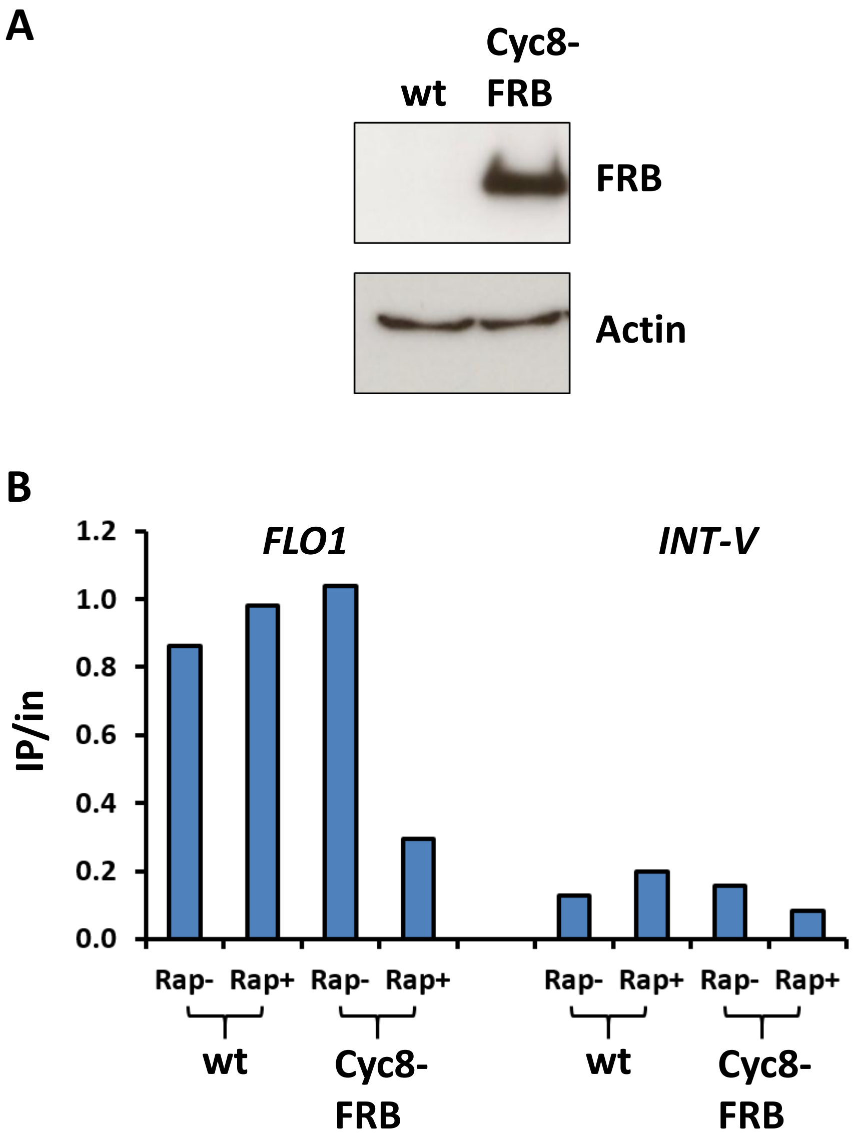
A
wt
Cyc8-FRB
FRB
Actin
B
IP/in
1.2
1.0
0.8
0.6
0.4
0.2
0.0
FLO1
INT-V
Rap- Rap+ Rap- Rap+
wt
Cyc8-FRB
Rap- Rap+ Rap- Rap+
wt
Cyc8-FRB

Fig. S2

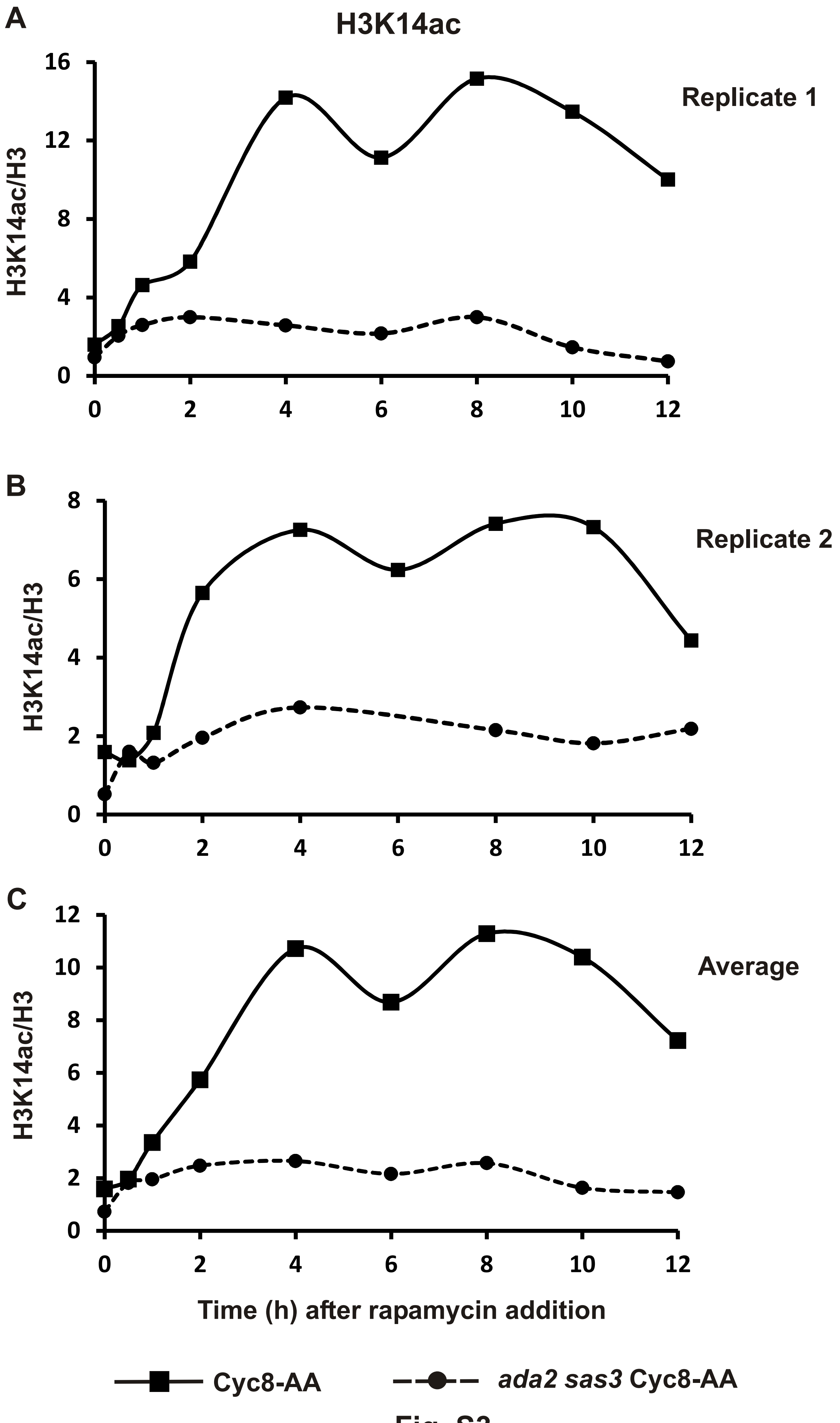

A
H3K14ac
Replicate 1
H3K14ac/H3
16
12
8
4
0
0 2 4 6 8 10 12
B
Replicate 2
H3K14ac/H3
8
6
4
2
0
0 2 4 6 8 10 12
C
Average
H3K14ac/H3
12
10
8
6
4
2
0
0 2 4 6 8 10 12
Time (h) after rapamycin addition
Cyc8-AA
ada2 sas3 Cyc8-AA

Fig. S3

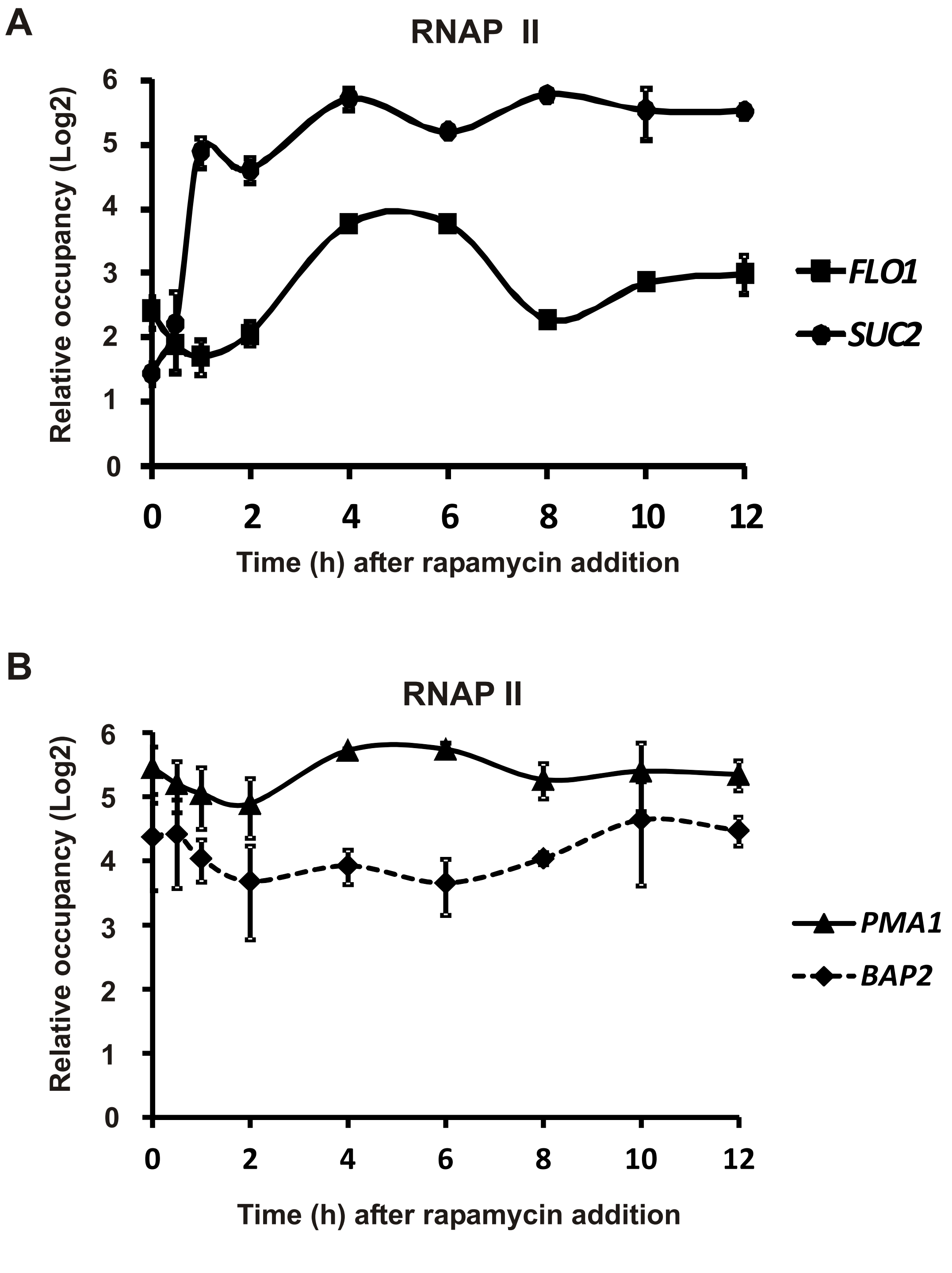
A
RNAP II
Relative occupancy (Log2)
0
1
2
3
4
5
6
0
2
4
6
8
10
12
Time (h) after rapamycin addition
FLO1
SUC2
B
RNAP II
Relative occupancy (Log2)
0
1
2
3
4
5
6
0
2
4
6
8
10
12
Time (h) after rapamycin addition
PMA1
BAP2

Fig. S4

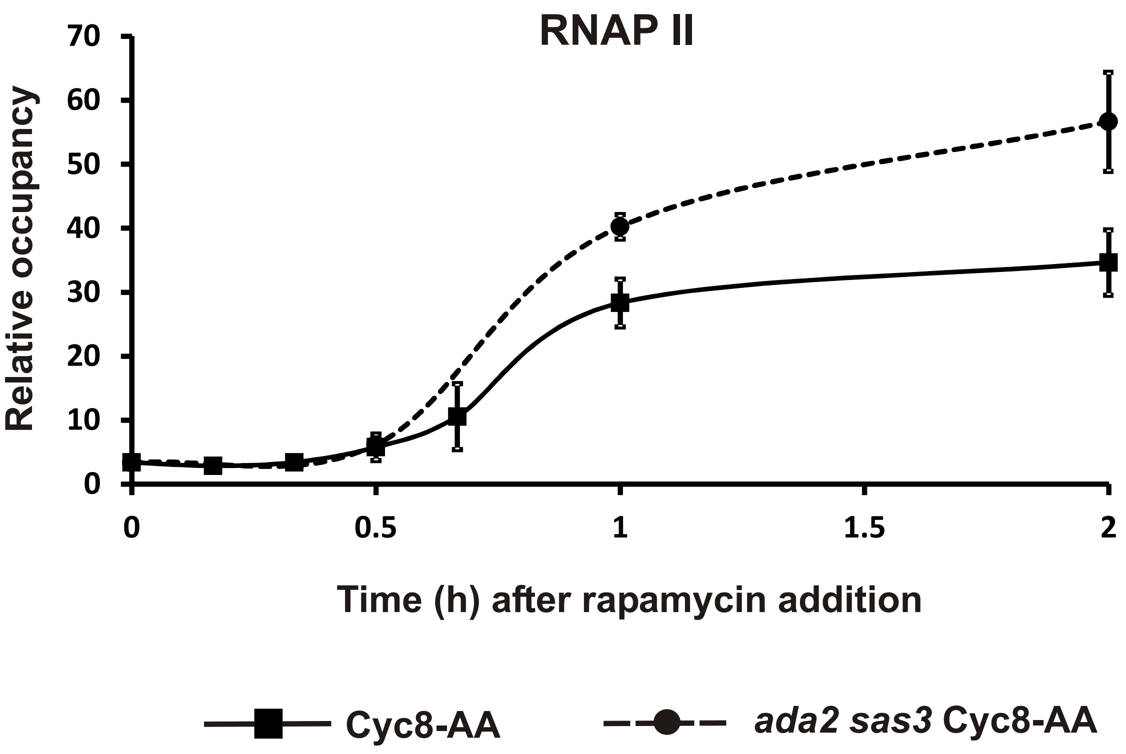

RNAP II
Relative occupancy
0
10
20
30
40
50
60
70
0
0.5
1
1.5
2
Time (h) after rapamycin addition
Cyc8-AA
ada2 sas3 Cyc8-AA

Fig. S5

Supplementary Figure Legends

Sas3 and Ada2(Gcn5)-dependent histone H3 acetylation is required for transcription elongation at the de-repressed *FLO1* gene

Michael Church[1], Kim C. Smith[1], Mohamed M. Mubarack[1], Sari Pennings[2] and Alastair B. Fleming [1,*]

Figure S1. Western blot analysis of whole cell lysates from the strains indicated were probed with antibodies against the Myc epitope to confirm the successful Myc-tagging of (A) Gcn5 and (C) Sas3 in the presence (Gcn5-Myc and Sas3-Myc) and absence of *CYC8* (*cyc8* Gcn5-Myc and *cyc8* Sas3-Myc). The Western blots were also probed with antibodies against Cyc8 to confirm Cyc8 abundance was unaltered in the Gcn5-Myc and Sas3-Myc strains. Untagged strains used as controls are also shown (wt, *gcn5* and *cyc8*) (B) Western blot analysis of H3K9ac in the strains indicated, confirm cellular levels of this histone modification are unaltered in the Gcn5-Myc tagged strains which indicate that Gcn5 function is not impaired by the Myc tag. (A-C) Actin served as a loading control in all blots. All proteins were of the expected sizes. (D) H3K9ac occupancy at the *FLO1* promoter. ChIP analysis of H3K9ac levels at the -585 bp region of the *FLO1* promoter. H3K9ac levels were normalised to H3 levels in the strains indicated. No difference in acetylation at the *FLO1* promoter was seen in *gcn5* mutants compared to wt; in both strains, Tup1-Cyc8 is present, and *FLO1* transcription is repressed. The results represent the mean from three independent experiments with bars depicting SEM.

Figure S2. Experiments to confirm the successful construction, and function, of the Cyc8 anchor-away (Cyc8-FRB) strain. (A) Western blots of protein lysates from wt and the Cyc8-FRB strain were probed with antibodies against the FRB epitope to confirm the Cyc8 protein was tagged with FRB; compare wt (untagged) and Cyc8-FRB (tagged). Actin is shown as a loading control. All proteins were of the expected sizes. (B) ChIP analysis to confirm loss of Tup1 occupancy occurs from the *FLO1* promoter in the Cyc8-FRB anchor-away strain after the addition of rapamycin. ChIP analysis of Tup1 occupancy at the *FLO1* promoter in the untagged (wt) and FRB-tagged Cyc8 (Cyc8-FRB) strains in the absence of Rapamycin (Rap-), and after growth for 2 hours in YEPD containing 1µg/ml rapamycin (Rap+). The Cyc8-FRB strains are referred to as 'Cyc8-AA' in the main text. Tup1 ChIP was performed to measure loss of the Tup1-Cyc8 complex following Cyc8-FRB anchor-away, as ChIP analysis using anti-FRB antibodies was technically poor and unreliable. Tup1 occupancy is shown as the ratio of IP/input at the target region 585 bp upstream of the *FLO1* transcription start site (*FLO1*), and at the intergenic region of chromosome V (*INT V*) which was used as a negative control.

Figure S3. Time course ChIP analysis measuring H3K14ac occupancy at the *FLO1* promoter in the Cyc8-anchor-away (Cyc8-AA) and *ada2 sas3* Cyc8-anchor-away (*ada2 sas3* Cyc8-AA) strains at the times indicated (h) after rapamycin addition. H3K14ac levels at the region 585 bp upstream of the *FLO1* transcription start site (*FLO1*) were normalised to *TEL-IV*, and are shown relative to histone H3 levels. The data shown in (A) and (B) are the independent biological replicates from which the average data was calculated, as shown in (C), and in Figure 7D. Note that although the trend is the same, the scales in the two graphs are different, thus precluding the addition of error bars.

Figure S4. Biphasic RNAP II occupancy at genes is not a consequence of Cyc8 anchor-away, whether the genes tested are prior targets of Tup1-Cyc8 occupancy (A), or not (B). (A, B) Time-course ChIP analysis measuring RNAP II occupancy at the *FLO1*, *SUC2, PMA1* and *BAP2* ORFs in the Cyc8-anchor-away (Cyc8-AA) strain at the times indicated (h) after rapamycin addition. RNAP II occupancy was normalised to levels at the *TEL-IV* region. The results represent the mean from three to four independent experiments with bars depicting SEM.

Figure S5. De-repression of *SUC2* after Tup1 anchor-away is not reduced by the deletion of the *ADA2* and *SAS3* genes. Time course ChIP analysis measuring RNAP II occupancy at the *SUC2* promoter in the Cyc8 anchor-away (Cyc8-AA) and *ada2 sas3* Cyc8 anchor-away (*ada2 sas3* Cyc8-AA) strains at the times indicated (h) after rapamycin addition. RNAP II occupancy was normalised to *the TEL-IV* regions. The results represent the mean from three independent experiments with bars depicting SEM.
